# Consensus recommendations for neurofibromatosis type 1 management in Asia Pacific

**DOI:** 10.1016/j.isci.2026.117002

**Published:** 2026-10-01

**Authors:** Yemima Berman, Beom Hee Lee, Anthony Pak-Yin Liu, Yoshihiro Nishida, Ee Shien Tan, Zhichao Wang, On behalf of APAC NF1 Expert Panel

**Affiliations:** 1 Kolling Institute, Northern Sydney Local Health District and the Faculty of Medicine and Health, The University of Sydney, Sydney, NSW, Australia; 2 Department of Paediatrics, Medical Genetics Center, Asan Medical Center, University of Ulsan College of Medicine, Seoul, Republic of Korea; 3 Department of Paediatrics and Adolescent Medicine, The University of Hong Kong, Hong Kong, China; 4 Department of Paediatrics, Hospital for Sick Children, Toronto, ON, Canada; 5 Department of Rehabilitation, Nagoya University Hospital, Nagoya, Japan; 6 Genetics Service, KK Women’s and Children’s Hospital, Singapore, Singapore; 7 Department of Plastic and Reconstructive Surgery, Shanghai Ninth People’s Hospital, Shanghai Jiao Tong University School of Medicine, Shanghai, China; 8 Neurofibromatosis Type 1 Center and Laboratory for Neurofibromatosis Type 1 Research, Shanghai Ninth People’s Hospital, Shanghai Jiao Tong University School of Medicine, Shanghai, China

**Keywords:** neurofibromatosis type 1, NF1, malignant peripheral nerve sheath tumor, MPNST, plexiform neurofibroma, ANNUBP, tumor surveillance

## Abstract

Neurofibromatosis type 1 (NF1) is a complex multisystem disorder requiring coordinated multidisciplinary care. This study aimed to develop consensus recommendations for NF1 management in the Asia-Pacific region. A modified Delphi process involving two survey rounds and a virtual meeting was conducted with experts from nine countries. A total of 141 statements were evaluated using a 9-point Likert scale with a 75% consensus threshold. Consensus was achieved across key domains including diagnosis, genetic testing, surveillance, and management. Experts emphasized the importance of multidisciplinary care, risk-adapted surveillance, and integration of genetic counseling and imaging in clinical practice. These findings provide regionally relevant guidance to support consistent and practical NF1 management across diverse healthcare systems.

## Highlights

• Multi-country Delphi consensus on NF1 management in Asia Pacific
• Provides region-specific recommendations across diagnosis and surveillance
• Emphasizes multidisciplinary and resource-adapted clinical care approaches
• Supports standardized decision-making across diverse healthcare systems

## Graphical abstract

**Figure:**
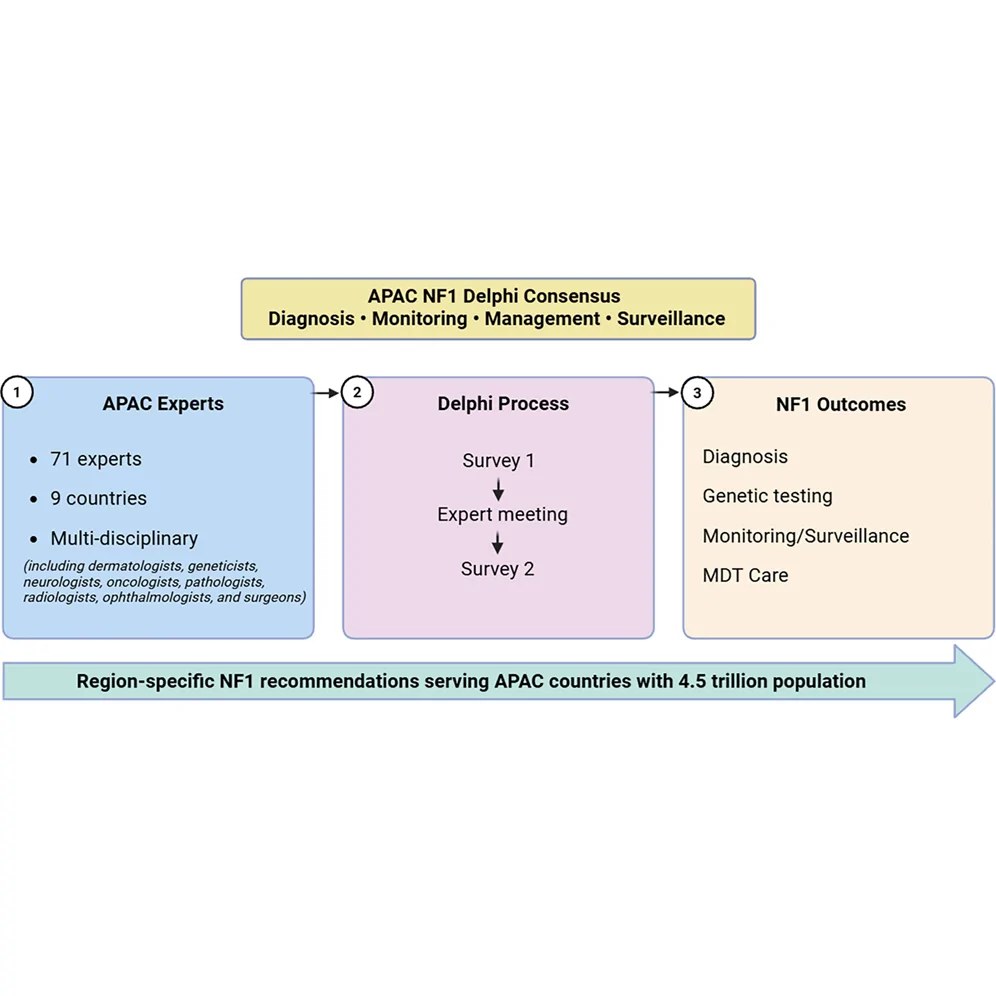

## Teaser

Health sciences; Medicine; Medical specialty; Internal medicine; Oncology; Public health

## Introduction

Neurofibromatosis type 1 (*NF1*), also known as von Recklinghausen’s disease, is an autosomal dominant genetic disorder that is characterized by the presence of tumors affecting the skin and central and peripheral nervous systems.^1^ *NF1* is estimated to have an incidence of approximately 1 in 2,500–3,000 births every year, whereas the global prevalence is estimated to be approximately 1 in 3,000–7,000.^2^^,^^3^ Obtaining accurate prevalence data for this condition is challenging due to the wide variability in the expression of *NF1*, differences in the populations studied, and the methods used to identify cases.^2^^,^^3^ Additionally, the lower prevalence observed in later adulthood may be attributed to higher mortality rates among individuals with *NF1* and the underreporting of adult patients.^2^

The clinical features of *NF1* include café-au-lait macules (CALMs), axillary and inguinal freckling, neurofibromas, and Lisch nodules.^4^ Neurofibromas are one of the most common and characteristic features of *NF1* and can be cutaneous neurofibromas (CNFs) or plexiform neurofibromas (*NF1*-PNs). CNFs are benign tumors that grow on nerves in the skin, while *NF1*-PNs are tumors of the peripheral nerves. *NF1*-PNs can be either symptomatic or asymptomatic, depending on their size and location. *NF1*-PNs carry a lifelong risk of malignant transformation into malignant peripheral nerve sheath tumors (MPNSTs), for which treatment options are limited, and mortality is high. The prognosis for patients who develop MPNST is generally poor, reflecting the aggressive nature of the tumor and the limited availability of effective therapies.

Benign *NF1*-PNs can transform to premalignant, atypical neurofibromas (ANs)/atypical neurofibromatous neoplasms of uncertain biological potential (ANNUBPs), approximately 8%–13% of individuals with *NF1* develop an MPNST, typically in young adulthood; risk varies with genotype, internal tumor burden, prior radiotherapy, and lesion dynamics. ANNUBP represents atypical lesions; malignant transformation risk should be judged case-by-case using integrated histologic and molecular criteria.^5^^,^^6^^,^^7^ ANs/ANNUBPs are characterized by at least two of the following features: cytological atypia, hypercellularity, loss of neurofibroma architecture, and an elevated mitotic index.^5^^,^^6^^,^^7^

*NF1*-PNs can also be classified based on their imaging characteristics, including their morphology, depth, and relationship to surrounding structures. Distinct nodular lesions (DNLs) are a type of peripheral nerve sheath tumor with a unique magnetic resonance imaging (MRI) appearance, compared with surrounding tissues.^5^^,^^6^ DNLs tend to grow faster than other types of *NF1*-PNs, develop later in the course, and may form a precursor to malignant transformation.^5^^,^^6^

Patients with *NF1* have been found to have a 2.7–4 times higher risk of developing malignancies compared with the general population, and their life expectancy is reduced by 8–20 years^7^ *NF1* typically has a wide range of clinical manifestations, which makes diagnosis, treatment, follow-up, and patient management challenging.

Considering that *NF1* is a complex disorder impacting multiple organ systems and potentially leading to serious complications, a collaborative, multidisciplinary approach among *NF1* specialists is crucial for consistent diagnosis and management. Recent advances in NF1 treatment, particularly the introduction of MEK (mitogen-activated protein kinase) inhibitors, have significantly improved the therapeutic landscape. In contrast, other targeted approaches such as mTOR inhibitors currently have limited clinical application in NF1 and require further investigation.^2^ Therefore, multidisciplinary collaborative guidelines and protocols for the diagnosis and treatment of *NF1* patients are important.

While established international NF-1 guidelines exist, these are largely developed for European and North American healthcare systems and do not address the unique needs of Asia-Pacific (APAC) region. Recent advances in the treatment landscape such as availability of targeted therapies and improved diagnostics necessitate updates to ensure relevance and accessibility. In order to address this, a panel of experts convened to harmonize evidence-based practices while considering cultural contexts and shared challenges, to complement Northern American/European guidelines by adapting them to APAC resource settings, not to replace them but to improve care across the region. Key considerations include differences in population structure and disease spectrum, potential ethnic variations in *NF1* manifestations, and common health-system barriers such as limited access to MRI, genetic testing, MEK inhibitors, centralized expertise, and fragmented care pathways. The approach adopted emphasizes applying international evidence in resource-limited settings through priority setting and defining minimum feasible surveillance, alongside fostering regional collaboration to overcome systemic challenges. To establish unified recommendations for the management of *NF1* across major APAC countries/regions including Australia, China, Hong Kong special administrative division of China, Japan, Malaysia, Singapore, South Korea, Thailand, and Taiwan, China, a modified Delphi consensus study with a scientific meeting was conducted to achieve consensus among geneticists, oncologists, neurologists, dermatologists, surgeons, ophthalmologists, radiologists, and pathologists in the region.

## Results

### Participants

Of the invited 71 participants, 51 (response rate: 71.8%) completed survey 1 and included dermatologists (*n* = 5), geneticists (*n* = 6), neurologists (*n* = 9), oncologists (*n* = 11), pathologists (*n* = 3), radiologists (*n* = 4), ophthalmologists (*n* = 3), and surgeons (*n* = 10). These participants were subsequently invited to a virtual participant meeting. In the meeting, 30/51 participants (58.8% attendance) were present. Following the discussion, a revised second survey was circulated to all 51 participants. Survey 2 was completed by 86.3% (*n* = 44/51) of the expert participants.

### Theme-wise consensus

Where original survey statements included more than one clinical decision, we analyzed and interpreted the results at the sub-component level to avoid misrepresenting consensus.

Table 1 summarizes the consensus recommendations reached during the study. In survey 1, participants reached a consensus on 53/97 closed-ended statements. The statements that did not reach consensus in survey 1 and eight statements added from the participant meeting formed survey 2. In survey 2, consensus was reached on 23/44 closed-ended statements.

**Table 1:** Comprehensive summary of consensus recommendations across delphi themes

**Theme 1. Diagnosis of NF1 and subtypes**	

**Category**	**Consensus recommendations**

Diagnosis of *NF1*	• Use revised Legius et al.^4^ criteria (≥ 6 typical CALMs, axillary/inguinal freckling, ≥ 2 neurofibromas, or ≥ 1 PN, OPG, ≥ 2 Lisch nodules or ≥ 2 choroidal abnormalities, distinctive osseous lesions, pathogenic *NF1* variant at 50% VAF). • Typical CALMs: round/oval, homogeneous brown, well-defined borders, > 5 mm (prepubertal). • Choroidal nodules: single lesions are supportive but not diagnostic; difficult to examine in young children; a single nodule cannot reliably differentiate *NF1* from Legius syndrome.
NF1-PN MRI Classification	• Classify using three domains: a. Morphology or internal structure: diffuse, fascicular/multi-nodular, predominantly diffuse or fascicular/multi-nodular, mixed patterns. b. Depth: superficial, deep, predominant forms, mixed patterns. c. Relationship with adjacent tissues: i. Interdigitating (tumors with ill-defined borders which penetrate and cannot be easily separated from adjacent tissues). ii. Circumscribed (tumors with smoothly defined borders which may compress and/or displace adjacent structures). iii. Predominantly interdigitating or circumscribed. iv. Mixed. NF1-PN can be classified as a “Distinct nodular lesion” to describe peripheral nerve sheath tumors with a characteristic MRI appearance.
Evaluation of NF1-associated MPNSTs	• For the evaluation of surgical specimens for MPNSTs, it is important that both the surgeon and the pathologist receiving the surgical biopsy specimen should carefully document surgical margins. • Multiple specimens should be collected during biopsy and should involve any regions of intra-tumoral heterogeneity.

**Theme 2. Genetic testing and counseling**	

**Category**	**Consensus recommendations**

Genetic counseling	• Genetic counseling by trained geneticists or supervised counsellors is essential for test selection, interpretation, and recurrence risk.
When to use multigene panels	• Use multigene panels when phenotype overlaps with other disorders. Panels should include: NF1, SPRED1, RASopathies (e.g., Noonan syndrome), CMMRD syndrome, KIT/KITLG (piebaldism), mismatch repair genes (MLH1, MSH6, PMS2, and MSH2), other relevant genes.
If sequencing is negative	• Perform deletion/duplication analysis (e.g., MLPA, SNP array, and OGM).
PGT	• PGT is suitable for couples wishing to avoid NF1. • Counseling should include all reproductive options. • Some couples may be unable to pursue PGT (maternal age, ovarian reserve, *de novo* variants, and inability to detect NF1 variants).

**Theme 3. Clinical manifestations, monitoring, and management**	

***3A. General NF1 follow-up***	

**Category**	**Consensus recommendations**

Non-complex vs. complex NF1	• NF1-specialized centers should monitor complex cases (such as those with a high burden of disease or features suspicious of malignancy). •Non-complex cases can be monitored locally by general practitioners/pediatricians who should be given appropriate guidelines for the management of NF1. Non-complex NF1 refers to individuals without clinically significant/asymptomatic internal plexiform neurofibromas and without clinical features associated with significant morbidity. External (superficial) plexiform neurofibromas may still lead to functional impairment, pain, disfigurement, or other complications, and therefore should not automatically be considered non-complex; instead, they require individual clinical assessment.
MDT	• Decision regarding surgery vs. pharmacological treatment for NF1-PN patients should be determined by a MDT or tumor board comprising surgeons, oncologists, and radiologists, among other applicable experts.

***3B. Children’s surveillance***	

**Category**	**Consensus recommendations**

Routine follow-up	• Age 0–5: clinical review every 6–12 months. • Age 6–10: annual review. • BP at every visit; HC measured through age 3.
Ophthalmology	• Annual ophthalmology until age 8. • Annual visual screening > 8 years.
Development and learning	• Developmental surveillance from birth. • Learning disability/ADHD screening at school entry.
Pain/QoL assessments	• Symptom-triggered assessments; annual pain scoring in children.
Cardiac and bone monitoring	• Echocardiography only if indicated or starting MEKi. • Monitor scoliosis progression (Adam’s forward bend test).
Vitamin D and Osteoporosis	• Vitamin D supplementation recommended. • Treat osteoporosis as per general population guidelines.

***3C. Imaging and tumor surveillance***	

**Category**	**Consensus recommendations**

Preferred modality	• MRI preferred over CT to avoid radiation. DWI can assist in distinguishing lesions with higher malignancy risk.
WBMRI	• Recommended at transition age for all patients. • Annual WBMRI for high-risk patients^a^: high tumor burden, NF1 microdeletion, prior radiotherapy. • Consider targetted imaging rather than WBMRI for symptomatic lesions • Across the patient lifespan, MRI timing follows a tiered approach based on age and risk. WBMRI is recommended once at the transition from childhood to adulthood as a baseline assessment of internal tumor burden. Annual WBMRI may be indicated for individuals with high-risk features such as high internal tumor burden, NF1 microdeletion, prior radiotherapy, rapidly growing PN or distinct nodular lesions. For patients whose PN can be adequately covered by local imaging, annual localized MRI is sufficient. Routine brain MRI for OPG is not recommended unless ophthalmologic assessment is unreliable or concerning.
Localized MRI	• Use for symptomatic PN, treatment response, or when regional assessment is sufficient.
PET-CT	• FDG-PET only when malignancy is suspected. Not for routine screening.

***3D. PN***	

**Category**	**Consensus recommendations**

When to intervene	• Rapid growth > 20% per year based on bidimensional (2D) measurements or impending morbidity = intervene. • Slow growth < 5% per year based on bidimensional (2D) measurements without symptoms = observe.
Treatment options	• Surgery is the only potentially curative option where feasible. • Partial surgery may be beneficial. • MEK inhibitors are recommended for symptomatic, inoperable PN or PN still symptomatic after partial surgery.
Radiotherapy	• Not recommended for benign PN due to risk of secondary malignancies and Moyamoya syndrome.

***3E. CNF***	

**Category**	**Consensus recommendations**

Clinical assessment	• Visual inspection + palpation of symptomatic lesions at every visit.
Treatment options	• Surgery, laser, electrodessication, radiofrequency ablation; other options emerging (topical MEKi, surfactant injections, alexandrite laser/pharmacological, and combination therapy).
Psychosocial support	• Psychological support recommended due to cosmetic and psychosocial burden.

***3F. MPNST***	

**Category**	**Consensus recommendations**

MPNST	• Complete surgical resection with negative margins is the only curative strategy for patients with MPNST. If the final pathology assessment indicates positive margins (excluding bone, nerves, or major vessels), thoughtful consideration should be given to conducting another surgical resection to ensure negative margins, as long as there is not an unacceptable impact on function • Adjuvant radiotherapy should be considered for cases where margins are close or there are microscopically positive margins involving bone, vital vessels, and nerves.

***3G. OPG***	

**Category**	**Consensus Recommendations**

Screening	• No routine MRI for asymptomatic children. • MRI only if ophthalmologic exam is unreliable or suspicious.
Treatment	• Treat only when there is progressive visual decline, symptomatic radiographic progression, or endocrine compromise.

***3H. Breast cancer screening***	

**Category**	**Consensus recommendations**

Screening cadence	• Start annual breast MRI at age 30. • If MRI unavailable: 3D mammography or 2D mammography + ultrasound. • Continue annual screening until age 50. • After age 50: follow national general-population screening guidelines.
Risk-reducing mastectomy	• Not recommended for NF1 patients without breast cancer.

***3I. Pheochromocytoma screening***	

**Category**	**Consensus recommendations**

When to screen	• Screen hypertensive NF1 patients, especially: age > 30, pregnant, or with paroxysmal hypertension, palpitations, sweating.^b^

***3J. NF1-associated GIST***	

**Category**	**Consensus recommendations**

Management	• Consider resection for symptomatic GIST or > 2 cm, due to risk of bleeding or metastasis.

**Theme 4. Child to adult transition**	

**Category**	**Consensus recommendations**

Transition planning	• Begin early; ensure patients are not lost to follow-up.
WBMRI at transition	• One-time WBMRI recommended to document tumor burden.
Ongoing imaging for high-risk patients	• Continue WBMRI or targeted MRI for patients with: high tumor burden, NF1 microdeletion, prior radiotherapy, DNLs, rapidly evolving PN.

Café-au-lait macules; CMMRD, constitutional mismatch repair deficiency; CNF, cutaneous neurofibroma; CT, computed tomography; F18-FDG-PET, fluorodeoxyglucose F18 positron emission tomography; MEK, mitogen-activated protein kinase; MLPA, multiplex ligation-dependent probe amplification; MPNSTs, malignant peripheral nerve sheath tumors; MRI, magnetic resonance imaging; *NF1*, neurofibromatosis type 1; *NF1*-PN, neurofibromatosis type 1 with plexiform neurofibromas; OGM, optical genome mapping; OPG, optic pathway glioma; PGT, preimplantation genetic testing.

a Painful/rapidly growing PN, DNLs, prior radiotherapy, and NF1 microdeletion genotype.

b Measuring plasma-free metanephrines or 24-h urinary fractionated metanephrines (with additional catecholamine assays as per local practice).

All statements rated by the participants are summarized in the Supplementary material (consensus in survey 1: Table S1; consensus in survey 2: Table S2; no consensus in either surveys: Figures S1–S4; and open-ended questions: Figures S5–S11).

For the diagnosis theme, 8/16 statements achieved consensus during the two surveys (survey 1: 4/16 and survey 2: 4/9). For the genetic testing and counseling theme, 11/17 achieved consensus during the two surveys (survey 1: 9/17 and survey 2: 2/8). Experts agreed that if initial sequencing results do not find a pathogenic or likely pathogenic variant, that further investigation for potential gene deletions should be considered. Techniques such as multiplex ligation-dependent probe amplification (MLPA), single-nucleotide polymorphism (SNP) array analysis, and optical genome mapping (OGM) were agreed to be effective methods for detecting gene loss (78% agreement).

For the clinical manifestation and monitoring theme, 51/62 statements achieved consensus during the two surveys (survey 1: 35/57 and survey 2: 16/25). Finally, for the child versus adult transition theme, 6/7 statements achieved consensus during the two surveys (survey 1: 5/7 and survey 2: 1/2).

Lack of consensus after both rounds of surveys can be attributed to APAC-specific factors, including availability, cost, and access to tests and procedures. When asked about utilizing *SPRED1* gene testing to differentiate between *NF1* and Legius syndrome, participants did not reach a consensus in both rounds of the survey. The participants also did not consider nevus anemicus and juvenile xanthogranuloma as additional diagnostic criteria for *NF1*.

## Discussion

A recent Delphi consensus from a Canadian expert group^8^ provides additional insights into NF1 management. Although there is substantial alignment between the Canadian and APAC recommendations in areas such as multidisciplinary care, tumor surveillance, and the role of MEK inhibitors, several differences reflect regional healthcare contexts.

In particular, the APAC recommendations place greater emphasis on adapting surveillance strategies based on resource availability, including access to MRI and genetic testing, whereas the Canadian consensus assumes broader access to specialized diagnostic and treatment infrastructure. These differences highlight the importance of contextualizing global evidence to regional healthcare systems and support the need for region-specific consensus recommendations.

### Diagnosis

For the diagnosis of *NF1*, the experts in the present study agreed with the criteria set forth by Legius et al.^4^ including the main criterion that six or more “typical” CALMs in pre-pubertal patients serve as the initial indication for suspecting or confirming a diagnosis. Interestingly, the experts participating in the study disagreed that choroidal abnormalities are a reliable indicator to differentiate *NF1* from Legius syndrome. In the revised *NF1* diagnostic criterion by Legius et al.,^4^ choroidal abnormalities were added as ophthalmologic criteria because of the high specificity and sensitivity for *NF1*,^4^ but they were not included as a separate criterion since isolated ophthalmologic findings, even if bilateral, are likely to reflect mosaic *NF1* rather than constitutional *NF1*. However, it should be noted that the participants in the study missed reaching consensus by a narrow margin (73% agreement). The presence of a single choroidal nodule is supportive of *NF1* but is not a stand-alone diagnostic criterion; in young children with only pigmentary findings, Legius syndrome remains a key differential, so *SPRED1* testing can be decisive when available. This likely explains why our panel narrowly missed consensus (73%) on their use to differentiate *NF1* from Legius.

Participants did not consider juvenile xanthogranuloma and nevus anemicus to be diagnostic features. The participants, during the scientific workshop, mentioned that due to heterogeneity of the disease and the different practices across countries, follow-up with the pediatrician after birth is not consistent across the APAC region. The participating experts further opined that there was concern surrounding misdiagnosis and unnecessary biopsy of lesions, which do not require resection. For the MRI-based classification of *NF1*-PN, the experts in the present study agreed upon the classification based on criteria including morphology or internal structure, depth, and relationship to adjacent tissues, as is consistent with the classification set forth by Fisher et al.^6^ This approach is consistent with internationally established NF1 tumor-surveillance recommendations, as in the EU and North American guidelines as well as the internationally recognized European Reference Network on Genetic Tumor Risk Syndrome (ERN GENTURIS) guidelines.^9^ The experts further highlighted that all three aspects should be considered together as a whole. Additionally, the experts agreed that DNLs should be used to describe peripheral nerve sheath tumors with a characteristic MRI appearance. DNLs are clearly defined, seem enclosed, measure ≥3 cm, do not exhibit the central target sign typical of classic PN, and may occur either within or outside of a PN.^5^^,^^6^ It should be noted that DNLs demonstrate varying growth patterns as compared with *NF1*-PN.^6^^,^^10^

For individuals who clearly meet the established diagnostic criteria for *NF1*, clinical diagnosis remains the primary approach. Genetic testing is generally reserved for purposes such as reproductive counseling, clarifying atypical or mosaic presentations, or distinguishing *NF1* from other RASopathies, including Legius syndrome. The recommendation for further testing, such as MLPA, SNP array, or conventional karyotype, apply primarily to patients with a strongly suggestive or borderline phenotype in whom initial sequencing is negative, as detecting gene deletions or structural variants can reduce false negatives and provide critical information for family planning.

### Genetic testing and genetic counseling

Distinguishing between *NF1* and other syndromes, such as Legius syndrome, in young children presenting solely with pigmented skin lesions can be challenging, particularly in the absence of a family history of *NF1* or other clinical features. Genetic testing is a useful tool for accurate diagnosis when available. Guidelines for the multidisciplinary diagnosis and treatment of *NF1* emphasize the importance of distinguishing *NF1* from similar syndromes at the time of diagnosis, including Legius syndrome, McCune-Albright syndrome, and Noonan syndrome.^11^^,^^12^ In the present study, there was agreement on the need for concurrent or stepwise genetic testing for Legius syndrome, constitutional mismatch repair deficiency (CMMRD) syndrome, and RASopathy syndromes such as Noonan syndrome. Although experts had differing opinions on the necessity of concurrent genetic testing for McCune-Albright syndrome, Russell-Silver syndrome, PTEN hamartoma tumor syndrome, chromosomal abnormalities, and piebaldism/familial hyperpigmentation, the possibility of these significant conditions occurring in patients suspected of *NF1* should be considered. In cases such as McCune-Albright syndrome and mosaicism, the need for tissue biopsy may have contributed to a lack of consensus. The experts opined that testing may not be feasible in some regions due to the availability and access to testing for these conditions. The experts reached a consensus that a multigene panel including at least *NF1*, *SPRED1*, RASopathy, CMMRD (inherited genetic syndrome caused by biallelic mutations), piebaldism (*KIT*, *KITLG*), mismatch repair (cellular process of correcting DNA replication errors) (*MLH1*, *MSH6*, *PMS2*, and *MSH2*), and other relevant genes may be considered when the phenotype cannot be differentiated from other disorders. This aligns with the current clinical practice of ordering multigene panels, which may help expedite diagnosis and may be more economical than ordering singular tests.

The experts in the study agreed that pre-implantation genetic testing (PGT) offers an option for couples to select embryos free from *NF1*, but timely counseling is essential to explore all reproductive choices. They acknowledged that some couples may be unable to pursue PGT due to factors like maternal age, reduced ovarian reserve, *de novo* inheritance, or challenges in detecting the underlying genetic cause. Additional reasons to not pursue PGT include cost and access to a care facility.^13^

### Clinical manifestation, management, and monitoring

The participants voted on various aspects of monitoring and management of *NF1*, including the subtypes, complications, and associated conditions, including *NF1*-PN, CNF, MPNSTs, and bone and neurological complications.

The participants of the current study agreed that children should undergo physical examinations. For children (0–10 years), conduct a general examination every 6–12 months from 0 to 5 years, then annually from 6 to 10 years. Check blood pressure at each visit. Measure head circumference at routine well-child visits through age 3 (then as indicated). Arrange annual ophthalmology until age 8 (earlier/more often if symptoms or unreliable testing). Provide developmental surveillance from birth and screen for learning disorders/ADHD when formal schooling begins. Use symptom-triggered pain/QoL tools rather than mandating annual scores in asymptomatic children. Perform Echocardiography only if clinically indicated or when initiating an MEK inhibitor (baseline and on-treatment cardiac/ophthalmic monitoring).^14^^,^^15^ In adults with non-complex NF1 (cutaneous manifestations only; without clinically impactful plexiform neurofibromas; stable ophthalmology, neurology, and orthopedics; normotension; and no significant pain), review every 2–3 years with clear safety-netting for new symptoms. For complex NF1 (e.g., with clinically impactful plexiform neurofibromas, progressive neurologic/orthopedic/ophthalmic issues, prior radiotherapy, high internal tumor burden, or NF1 microdeletion), schedule at least annual clinical review with WBMRI or targeted imaging as indicated. This approach aligns with international NF1 tumor-surveillance guidance by recognizing differences in disease complexity and avoiding one-size-fits-all schedules.

The majority of the patients with *NF1* develop CNFs. The experts in the present study agreed that visual inspection and palpation of CNFs should be conducted at every visit. Treatment options for CNFs may include surgical approaches (such as surgery, laser, electrodesiccation, or radiofrequency ablation), as well as emerging pharmacological and combination therapy options, including topical MEK inhibitors and other investigational treatments. These opinions align with the guidelines set forth by the ERN GENTURIS.^16^

For transition (late adolescence) – plan an annual clinical review and a one-time whole-body MRI (WBMRI) (where available) to quantify internal tumor burden and document DNLs. Thereafter, tailor imaging – prioritize closer follow-up in those with high internal tumor burden, NF1 microdeletion, prior radiotherapy, rapidly changing PN, or DNLs. Patients with higher internal tumor burden are predisposed to developing malignancies, specifically the development of MPNSTs, with peak standardized mortality in young adulthood.^7^ Hence, it is important that the patient should be monitored at least during the transition from childhood to adulthood.^16^^,^^17^ The participating experts in the present study recommended that WBMRI should be conducted at the transition from childhood to adulthood. This recommendation aligns with those set forth by the ERN GENTURIS guidelines and the expert consensus on full course management of *NF1*-PN in China.^16^^,^^18^ WBMRI refers to a coronal, multi-station MRI protocol covering head, spine, trunk, and extremities. However, considerable variation between protocols exists due to a lack of established international standards. High internal tumor burden refers to numerous and/or voluminous internal neurofibromas on WBMRI, often with DNLs. When feasible, quantify total internal tumor volume and document distribution; higher volumes and DNLs have been associated with increased clinical risk and are useful to guide follow-up intensity. No universal numeric cut-off exists; local volumetry and cohort distributions should be used to stratify low vs. high burden.

ERN GENTURIS guidelines indicate that for symptomatic *NF1*-PN, surgery is the only treatment that has the potential to cure the tumor, and *NF1*-PN surgery should be considered.^16^ However, the risks of bleeding and residual scarring should be considered, and complete resection of the tumor is often not possible.^19^ Consistent with the evidence, the participants in the current study agreed that complete surgery is only rarely an effective option for symptomatic *NF1*-PN. However, experts agreed that partial surgery may be beneficial for patients in some cases. Radiotherapy is not recommended for benign *NF1*-PN due to the risk of secondary malignancies. The participants further agreed that MEK inhibitor is a treatment option for symptomatic, inoperable *NF1*-PN, and for those who remain symptomatic despite partial resection.^20^ As of 2026, selumetinib has US FDA and EU approval – marketing authorization (without conditional approval) for adults and pediatric patients (≥ 3 years) with symptomatic, inoperable PN; mirdametinib has US FDA approval for adults and children ≥ 2 years with symptomatic, inoperable PN.^21^^,^^22^^,^^23^

A summary of key clinical trials evaluating MEK inhibitors, including selumetinib, mirdametinib, trametinib, binimetinib, and tunlametinib, in NF1-associated plexiform neurofibromas is presented in Table 2.

**Table 2:** Key clinical trials of MEK inhibitors in NF1-PN

Trial/Study	Population	Study type	Key findings
Selumetinib Phase 1/2 pediatric Trial^24^	Children with inoperable NF1-PN	Phase 1/2 clinical trial	Demonstrated long-term safety and efficacy; sustained tumor shrinkage and improvement in pain and quality of life.
NEJM 2020 Selumetinib trial (Gross et al.^25^)	Children with inoperable PN	Randomized pediatric trial	Significant and durable volume reduction; clinical improvement in symptoms.
Chinese Phase 1 trial (Wang et al.^26^)	Chinese adults and children	Phase 1 open-label	Demonstrated safety, pharmacokinetics, and efficacy consistent with international experience.
Korean Real-World Study (Kim et al.^27^)	Adults and pediatric patients	Observational	Confirmed safety and tumor-shrinkage efficacy in real-world APAC setting.
KOMET Phase 3 International Trial^28^	Adults with inoperable NF1-PN	Multicenter, randomized, placebo controlled	Demonstrated significant tumor shrinkage and clinical benefit in adults; first high-quality adult RCT.
Selumetinib 2025 Adult Phase 2 Trial (Gross et al.^29^)	Adults with inoperable PN	Phase 2 trial	Reported durable responses and tolerability in adults.
Mirdametinib ReNeu Phase IIb Trial^30^	Adults and children with symptomatic PN	Phase IIb pivotal trial	Reported tumor shrinkage, pain reduction and functional improvements across ages.
TRAM-01 (Sadat Kiaei et al.)^31^	Pediatric patients with NF1-associated plexiform neurofibromas	Phase 2 clinical trial	Demonstrated meaningful tumour reduction and acceptable safety profile, supporting trametinib as an additional MEK inhibitor option in NF1-associated PN.
PNOC010 / NF108-BINI Binimetinib Study^32^	Children and adults with NF1-associated PN	Phase 2 open-label trial	Demonstrated clinical activity and tumour volume reduction in patients with inoperable plexiform neurofibromas.
Tunlametinib Phase IIa Trial (Wang et al.)^33^	Adults with inoperable NF1-associated plexiform neurofibromas	Phase IIa open-label trial	Selumetinib represents the first MEK inhibitor to

Selumetinib represents the first MEK inhibitor to receive regulatory approval for the treatment of symptomatic, inoperable NF1-associated plexiform neurofibromas, marking a major therapeutic milestone and shifting management beyond surgery alone. Although initially authorized for pediatric use, subsequent adult studies including large international trials have demonstrated meaningful tumor shrinkage and clinical benefit in adults as well.^24^^,^^25^^,^^28^^,^^30^^,^^34^ Emerging evidence also supports the use of other MEK inhibitors in NF1-associated plexiform neurofibromas. The recent TRAM-01 Phase II study demonstrated tumour reduction and an acceptable safety profile in paediatric patients treated with trametinib.^31^ Additional studies evaluating binimetinib,^32^ tunlametinib, and FCN-159 have similarly reported meaningful tumour reduction and encouraging safety profiles, further supporting the role of MEK inhibition in NF1 management.^33^^,^^35^

Mirdametinib, now FDA-approved for both adults and children ≥ 2 years, further expands the role of MEK inhibition as a core treatment option for PN across the age spectrum.^30^ Together, these agents establish MEK inhibition as the first effective systemic therapy class for *NF1*-PN, with safety monitoring (cardiac and ophthalmic) remaining an essential component of care.

Although a majority of the experts agree that MEK inhibitors can be considered for *NF1*-PNs that remain symptomatic after partial surgery, the available safety and efficacy data are relatively limited. Furthermore, the current focus on volumetric reductions as an outcome may underestimate the response in patients who have significant symptomatic relief and improvement in quality of life despite no substantial tumor reduction. The utility of MEK inhibitors as adjuncts to surgical resection in patients amenable to complete resection remains to be further investigated. Finally, the optimal target patient population and duration of treatment remain uncertain. Typically, the treatment is continued as long as benefits last; however, the costs associated with prolonged treatment need to be assessed.^36^

We adopt the 2025 integrated diagnostic framework for NF1-associated peripheral nerve sheath tumors. ANNUBP can be supported by CDKN2A/B inactivation, whereas MPNST is supported by PRC2 (SUZ12/EED) or TP53 inactivating alterations and/or significant aneuploidy. We avoid the term “low-grade MPNST,” preferring “ANNUBP with increased proliferation” when molecular criteria for MPNST are not met.

MPNSTs can develop anywhere in the body but are most commonly found near major nerve plexuses and roots. The primary goal in treating MPNST is to achieve an R0 resection (negative margins post-resection). However, this can be challenging due to the tumor’s proximity to vital anatomical structures. Despite these challenges, aiming for an R0 resection is crucial upon diagnosis, as incomplete resection often results in a high recurrence rate, with recurrence being almost inevitable. This recurrence typically leads to tumor regrowth and is associated with a poor prognosis for patients.^37^^,^^38^ The experts in the present study reached a consensus that negative margins are the goal for patients with MPNST, and thoughtful consideration should be given when positive margins are identified post-resection and repeat resection is feasible. For the patients in whom margins are close or there are microscopically positive margins involving bone, vital vessels, and nerves, the experts agreed that radiotherapy should be considered. Radiotherapy is most beneficial for patients with large tumors (e.g., >5 cm), high-grade malignancies, and/or those who have positive surgical margins following resection. However, caution must be exercised as radiation is a documented risk factor for the development of MPNSTs in patients with *NF1*.^37^ According to a retrospective, population-based study conducted by Huang et al., although radiotherapy was linked to improved survival rates in advanced MPNST cases, the risk of mortality significantly increased for secondary MPNST.^39^

The experts in this study recommended that ophthalmological assessment should be repeated annually until the age of 8 years and into adulthood. However, in this study, experts did not reach a consensus on considering brain MRI for optic pathway glioma (OPG) screening. There was also no consensus on the statement that caution should be taken in recommending a brain MRI for OPG screening. Imaging for OPGs with MRI should be performed in individuals where an ophthalmological examination suggests the presence of an OPG. Additionally, it should be considered in children who have repeated inconclusive or unreliable ophthalmological exams, such as those due to age or attention deficits.^16^^,^^40^^,^^41^

Consensus was not to perform routine brain MRI screening in asymptomatic children with NF1. Treatment of OPG was recommended when there is documented progressive visual decline, radiographic progression with symptoms, or hypothalamic-endocrine compromise (e.g., precocious puberty). Many NF1-OPGs remain stable; observation is appropriate in stable disease. Vincristine-carboplatin remains a standard first-line regimen; targeted approaches are emerging, but long-term visual outcomes are still being defined.^42^^,^^43^^,^^44^^,^^45^ Consistent with the evidence, the experts in this Delphi study disagreed on considering weekly vinblastine monotherapy as an alternative to first-line treatment, vinorelbine or temozolomide as second-line chemotherapy, and treatment with bevacizumab with or without other chemotherapy agents.

The experts in the study agreed that annual screening in patients for breast cancer in *NF1* patients should start after age 30, with breast MRI as the primary recommended screening method. If MRI is unavailable, 3D tomosynthesis mammography or 2D mammography combined with ultrasound is the next best alternative. Breast cancer screening should continue annually until age 50. Risk-reducing bilateral mastectomy should not be performed in *NF1* patients without breast cancer, as there is insufficient evidence to support its benefit. ERN GENTURIS guidelines, French national guidelines, and NCCN (National Comprehensive Cancer Network) guidelines recommend that, after 50 years of age, breast cancer screening for women with *NF1* should be according to national guidelines for the general population.^16^^,^^46^^,^^47^

### Child to adult transition

ERN GENTURIS guidelines recommend surveillance for OPG, *NF1*-PN, gliomas, and brain tumors and assessment of internal tumor burden at the transition from childhood to adulthood. The experts in the present study align with the recommendations by ERN GENTURIS. The guidelines further recommend that in the absence of internal neurofibromas detected on WBMRI at the transition age to adulthood, clinical assessment only is required.^16^

### Limitations of the study

Our study has several strengths, including its large-scale design and the involvement of a multi-disciplinary panel of participants, which encompassed specialists in genetics, oncology, neurology, dermatology, surgery, ophthalmology, radiology, and pathologists. To our knowledge, this is the first multi-country APAC Delphi consensus spanning nine countries, designed to adapt internal evidence to APAC health system realities. This study explored various themes in *NF1* diagnosis and management, and achieved a good response rate, indicating strong engagement from the expert community. Additionally, the use of an independent facilitator and the provision of anonymous, supervised feedback ensured the robustness and reliability of the process.

There are certain limitations to our study. The results of the Delphi study can be considered expert opinions, and other expert panels may not reach the same conclusions. Furthermore, the results obtained from this study have temporal validity, as they may change over time, and participant views were not unanimous on all questions. In addition to psychologists and psychiatrists, other key healthcare professionals such as specialist nurses and pharmacists were not included in this Delphi panel. These professionals play an important role in patient education, long-term follow-up, medication management, treatment adherence, and supportive care, particularly within theme 3 (clinical manifestations, monitoring, and management). Their absence may limit the comprehensiveness of recommendations related to multidisciplinary care delivery. This reflects current practice in many APAC settings, where NF1 care is often led by physician specialists, and structured multidisciplinary teams (MDTs) incorporating allied health professionals are less consistently established. Future consensus initiatives should aim to include a broader range of healthcare providers to better capture holistic patient care perspectives. This absence is attributable to the fact that in many APAC settings, mental-health professionals are not yet routinely integrated into *NF1* multidisciplinary clinics, and both service structures and patient priorities remain more focused on tumor control, pain, and functional issues than on formal psychological or psychiatric assessment. However, the psychological burden of the disease is substantial and should form a focus of future consensus studies. We acknowledge that some survey items were compound; we therefore disaggregate results and highlight where partial agreement applied. Our novelty lies in APAC adaptation, bridging international evidence with resource-variable settings rather than replacing existing US/EU guidelines. As a result, these findings should be interpreted rationally, and further work will be needed to clarify the areas of uncertainty detected in the results.

In summary, the findings of this Delphi study provide important guidance for NF1 management across ages and across the spectrum of disease manifestations and severity. The expert recommendations reflect meaningful advances achieved in NF1 monitoring and care in recent years, including clearer surveillance pathways, improved use of imaging technologies, incorporation of genetic testing, and recognition of the chronic and multisystem nature of NF1. The results demonstrate broad alignment with established international guidelines, including the ERN GENTURIS tumor-surveillance recommendations, for the vast majority of statements.

The experts also emphasized crucial points in the NF1 care pathway, such as the transition from pediatric to adult services, where tailored intervals for clinical review and imaging based on individual disease burden are essential. Finally, both pediatric and adult experts strongly highlighted that a MDT approach is necessary and desirable across the APAC region to ensure that NF1 patients receive comprehensive, coordinated, and high-quality care.

Consensus was reached on several key concepts related to *NF1* diagnosis and management, including clinical diagnostic criteria for *NF1*, classification of *NF1*-PN and MPNSTs. The experts also agreed on the role of genetic counseling, testing, and differentiating between various overlapping genetic syndromes. The experts further highlighted the importance of a multidisciplinary approach toward the management of *NF1* and proposed that surgeons, oncologists, and radiologists often form the core of these MDTs, but other specialties may be involved or lead these MDTs depending on local expertise. We propose a life-stage framework: every 6–12 months (0–5 years), annually (6–10 years), annual transition with one-time WBMRI (where available), and in adulthood every 2–3 years for non-complex vs. at least annually for complex NF1. The divergent opinions of the experts surrounding genetic testing and clinical monitoring were noted, including monitoring for certain genetic syndromes and measures such as WBMRI, which diverge from the published guidelines.

## Resource availability

### Lead contact

Further information and requests should be directed to the Lead Contact, Dr. Zhichao Wang (wangzhichao@sh9hospital.org.cn).

### Materials availability

This study did not generate new unique reagents or materials.

### Data and code availability

All data generated or analyzed during this study are included in this published article and its Supplementary Information files. Additional information is available from the corresponding author upon reasonable request.

## Consortia

APAC NF1 Expert Panel Members (Delphi Participants).

The authors acknowledge the following experts who participated in the Delphi consensus process:

**Australia**

Dr. Hannah North, RNSH Hospital, Australia

Dr. Jordan Hansford, Department of Pediatric Oncology, Women’s and Children’s Health Network, Women’s and Children’s Hospital, Australia

Dr. Tony Pennington, Department of Pediatric Plastic Surgery, Sydney Local Health District, Royal Prince Alfred Hospital

**China**

Dr. Jian Wang, Department of Pathology, Fudan University Shanghai Cancer Center, China

Dr. Li Qingfeng, China Multicenter Treatment Collaborative Group for Type I Neurofibromatosis, Shanghai Ninth People’s Hospital, Shanghai Jiao Tong University School of Medicine, China

Dr. Peiquan Zhao, Department of Ophthalmology, Xin Hua Hospital Affiliated to Shanghai Jiao Tong University School of Medicine, China

Dr. Pi-Nan Liu, Department of Neurosurgery, Beijing Tiantan Hospital of Capital Medical University, China

Dr. Weiqing Tang, Department of Radiology, Shanghai Ninth People’s Hospital, Shanghai Jiaotong University School of Medicine, China

Dr. Xinghua Gao, Department of Dermatology, The First Hospital of China Medical University, China

Dr. Yiwei Wu, Department of Neurosurgery, Shanghai Ninth People’s Hospital, Shanghai Jiaotong University School of Medicine, China

Dr. Zezhang Zhu, Department of Spine Surgery, The Affiliated Drum Tower Hospital of Nanjing, University Medical School, China

Dr. Zhimiao Lin, Department of Dermatology, Dermatology Hospital, Southern Medical University, China

Dr. Zhong-Ping Chen, Department of Neurosurgery, Sun Yat-sen University Cancer Center, China

Dr. Xin Ni, Department of Head and Neck Surgery, Beijing Children’s Hospital of Capital Medical University, China

Dr. Man-mei Long, Department of Pathology, Shanghai Ninth People’s Hospital, Shanghai Jiaotong University School of Medicine, China

**Hong Kong Special Administrative Region of China**

Dr. Eva Fung, Department of Pediatric Neurology, The Chinese University of Hong Kong, Hong Kong

Dr. Karen Yam, Department of Pediatric Neurology, The Chinese University of Hong Kong, Hong Kong

**Japan**

Dr. Keita Terashima, Department of Pediatric Neuro-Oncology, National Center for Child Health and Development, Japan

Dr. Kunihiro Ikuta, Department of Orthopedic Surgery, Nagoya University Graduate School of Medicine, Japan

Dr. Monji KOGA, Department of Dermatology, Faculty of Medicine, Fukuoka University, Japan

Dr. Robert Nakayama, Department of Clinical Oncology, Orthopedics, Keio University Hospital (Sarcoma and Melanoma Center), Japan

Dr. Toshiki Takenouchi, Department of Pediatrics, Neurology, Keio University Hospital (Craniofacial Center), Japan

Dr. Yuichi Yoshida, Department of Dermatology, Tottori University Hospital, Japan

**Malaysia**

Dr. Poorani Anandakrishnan, Department of Pediatric Neurology, Pantai Hospital Kuala Lumpur, Malaysia

Dr. Foo Jen Chun, Department of Paediatric Hematology-Oncology, University of Malaya Medical Centre, Malaysia

Dr. Hamidah Alias, Department of Paediatric Hematology-Oncology, UKM Specialist Children’s Hospital, Malaysia

**Singapore**

Dr. Enrica Tan, Department of Pediatric Oncology, KKH, Singapore

Dr. Joanne Ngeow Yuen Yie, Department of Oncology-Medical, Genetics, National Cancer Center Singapore, Singapore

Dr. Miriam Santiago Kimpo, Department of Pediatric Hematology-Oncology, NUH, Singapore

Dr. Saadia Zohra Farooqui, Department of Ophthalmology, Sing Health, Singapore

**South Korea**

Dr. Byung Joo Lee, Department of Ophthalmology, Asan Medical Center, South Korea

Dr. Eun Na Kim, Department of Pathology, Seoul National University College of Medicine, South Korea

Dr. Hee Mang Yoon, Department of Radiology, Asan Medical Center, South Korea

Dr. Heyry Kim, Department of Pediatrics, Asan Medical Center Children’s Hospital, South Korea

Dr. Jeong Eun Kim, Department of Adult Oncology, Asan Medical Center, South Korea

Dr. Jung Min Ko, Department of Pediatrics (Geneticist), Seoul National University Hospital, South Korea

Dr. Pyeong Hwa Kim, Department of Radiology, Asan Medical Center, South Korea

Dr. Yeong-Bae Son, Department of Pediatrics (Geneticist), Ajou University Hospital, South Korea

Dr. Jung Woo Han, Department of Pediatric Hematology and Oncology, Yonsei University Health System, Yonsei University College of Medicine, South Korea

Dr. Hyun-young Kim, Department of Surgery, Seoul National University College of Medicine, South Korea

**Taiwan** **, China**

Dr. I-Jun Chou, Division of Pediatric Neurology, Chang Gung Children’s Hospital, and Chang Gung Memorial Hospital, Chang Gung University College of Medicine at Linkou, Taoyuan, Taiwan, China

Dr. Ming-Jen Lee, Department of Neurology, National Taiwan University Hospital, Taiwan, China

Dr. Pi-Chuan Fan, Department of Pediatric Neurology, National Taiwan University Hospital, Taiwan, China

Dr. Tu-Hsueh Yeh, Department of Neurology, Taipei Medical University Hospital, Taiwan, China

Dr. Yen-Lin Liu, Department of Neurology, Taipei Medical University Hospital, Taiwan, China

**Thailand**

Dr. Kanya Suphapeetiporn, Department of Genetics, King Chulalongkorn Memorial Hospital, Thailand

Dr. Piti Techavichit, Department of Pediatric Hematology-Oncology, King Chulalongkorn Memorial Hospital, Thailand

Dr. Rattanavalai Nitiyarom, Department of Pediatric Dermatology, Siriraj Hospital, Mahidol University, Bangkok, Thailand

Dr. Surachai Likasitwattanakul, Department of Pediatric Neurology, Siriraj Hospital, Mahidol University, Bangkok, Thailand

Dr. Thipwimol Timaroon, Department of Pediatric Genetics, Ramathibodi Hospital, Thailand

Dr. Wuttichart Kamolvisit, Department of Pediatric Genetics, King Chulalongkorn Memorial Hospital, Thailand

All listed expert panel members contributed to the Delphi surveys and consensus discussions but did not meet the journal criteria for authorship.

Generative AI and AI-assisted technologies were not used in the preparation of this work.

## Acknowledgments

This study (data collection, analyses, medical writing, and publication support) was sponsored by Alexion, AstraZeneca Rare Disease as part of an alliance between AstraZeneca and Merck Sharp and Dohme, LLC, a subsidiary of Merck & Co., Inc., Rahway, NJ, USA (10.13039/100030732MSD). Alexion provided a review of the manuscript but did not influence the data, analysis, or interpretation. The authors were involved in the development of this publication and retain final authority, including the choice of journals for publication. The authors thank Ms. Priyanka Barathe and Dr. Neha Deshpande from Trinity Life Sciences (Singapore), who provided medical writing and editorial support for this manuscript. They also would like to extend their heartfelt gratitude to the participating experts in this study for their invaluable contributions to the project.

## Author contributions

All authors had full access to all the data in the study and contributed to conceptualization, methodology, data curation and analysis, validation and visualization, writing, reviewing, and editing of the initial and final drafts.

## Declaration of interests

The authors received support for medical writing from Trinity Life Sciences (Singapore) which was funded by Alexion, AstraZeneca Rare Disease.

Y.B., A.P.Y.L., and E.S.T. declare no other conflicts for this work.

B.H.L. has received honoraria, grants, consulting fees, travel support, and has served on advisory boards from AstraZeneca.

Z.W. has received grants from National Natural Science Foundation of China and Science and Technology Commission of Shanghai Municipality.

Y.N. has received consulting fees, honoraria, and grants from Alexion Pharma; served on advisory board for Alexion Pharma; and holds a leadership role in the Japanese Society of Recklinghausen Disease.

## STAR★Methods

### Experimental model and study participant details

Seventy-one expert clinicians from nine APAC countries/regions, with recognized expertise in *NF1* management were invited to participate. Participants for the study were selected using predefined eligibility criteria as part of a purposive sampling approach rather than through random sampling, to ensure inclusion of clinicians with substantial expertise in NF1 management. This approach is consistent with established Delphi methodology, where expert input is prioritized over population representativeness. Efforts were made to ensure broad geographic representation across the Asia-Pacific region, including Australia, China, Hong Kong SAR, Japan, Malaysia, Singapore, South Korea, Thailand, and Taiwan, China.

In addition, disciplinary diversity was ensured by including specialists from genetics, oncology, neurology, dermatology, surgeons (including neurosurgeons, plastic and orthopedic surgeons), radiology, ophthalmology, and pathology. This multidisciplinary composition was intended to reflect the complex, multisystem nature of NF1 and to capture diverse clinical perspectives across different healthcare settings. Collectively, this approach enhances the relevance and applicability of the consensus recommendations across heterogeneous APAC healthcare systems.

The selection criteria included.

• board-certification in a relevant specialty (e.g., clinical genetics, oncology, neurology, dermatology, surgery, radiology, ophthalmology, or pathology);
• currently practising in an *NF1* multidisciplinary clinic or recognised *NF1* referral center.

Participants meeting the pre-defined eligibility criteria were invited to participate in the study using individual emails. Participants who completed Survey-1 were invited to join a virtual participant meeting and participate in Survey-2. Participation in Survey-2 was independent of the participant’s meeting attendance.

A steering committee (the author group) comprising six experts (nominated based on their long-standing clinical and academic expertise in *NF1*, leadership roles in *NF1* services or networks, and representation of different regions and key specialties) endorsed the questionnaire, moderated the participant meeting and approved the outcomes of the study.

All participants provided informed consent prior to participation. As this was a non-interventional physician survey, formal ethical approval was not required.

### Method details

#### Study design

This study employed a modified Delphi technique, which included two rounds of cross-sectional surveys and a virtual participant meeting among experts in the APAC region for the development of consensus recommendations for the management of *NF1* in the APAC region.

Cross-sectional surveys were conducted between August 2024 and January 2025, while a participant meeting was held in October 2024. A research team developed the survey questionnaire, which was administered by an independent vendor using Decipher software (version Compact = 153). Responses were analyzed after each survey round using Microsoft Excel. The percentage of participants selecting each category of responses was calculated, and consensus was determined using a pre-determined threshold. All participants’ information during the surveys was kept confidential and anonymous. Since this was a non-interventional physician survey, no formal ethical approval was required.

Some survey statements inadvertently combined more than one clinical decision within a single item (for example, different tumor types, multiple surveillance intervals, or several PN characteristics grouped together). Because participants could not respond to each component separately, we handled this by disaggregating these items during analysis. In the Results and Supplement, we report subcomponent-level interpretation and indicate where only partial agreement applied. This approach does not change the original voting percentages but clarifies how the responses should be interpreted.

#### Development of the survey questionnaire

Survey-1 was developed from key statements drawn from a targeted literature review focused on the objectives of this Delphi study. The objectives included understanding the current practices followed in the region and recommending appropriate modifications to optimise outcomes in *NF1* patients.

A targeted literature search was conducted in the PubMed database to inform the development of the Delphi questionnaire. The search strategy included combinations of keywords such as ‘Neurofibromatosis type 1’, ‘NF1’, ‘plexiform neurofibroma’, ‘MPNST’, ‘guidelines’, ‘consensus’, ‘diagnosis’, ‘management’, and ‘surveillance’, using Boolean operators (AND/OR) to refine results.

The search included publications up to June 2024 and was limited to studies conducted in human populations and published in English. Eligible studies included clinical trials, observational studies, review articles, and existing clinical guidelines relevant to NF1 diagnosis and management.

Titles and abstracts were screened for relevance, followed by full-text review where appropriate. In addition, gray literature sources, including conference proceedings, expert consensus statements, and guideline documents, were reviewed to supplement the published evidence.

Key findings from the literature were synthesised by the study team and used to develop the initial set of Delphi statements. Further methodological details are provided in the Supplementary Material.

A questionnaire with 141 statements was developed to address the following themes.

• Diagnosis (n = 17)
• Genetic testing and genetic counselling (n = 17)
• Clinical manifestation, management and monitoring (n = 93)
• Child to adult transition (n = 14)

Although clinical manifestations could also be grouped with clinical features and diagnosis, the study team intentionally retained them as a separate theme. This structure reflects how the Delphi questionnaire was originally developed and ensures that statements are not reorganized post-hoc. Clinical manifestations often influence management and monitoring decisions beyond initial diagnosis; therefore, maintaining them as an independent theme allowed clearer categorization and alignment with the way experts evaluated the statements.

#### Delphi process

The first round of the Delphi survey was shared electronically through individual e-mails to the participants and completed online between August and September 2024. Fifty-one participants from the first round of the survey were invited to an online, virtual meeting to review the survey results and to share their expert views, focusing primarily on statements that did not achieve consensus in survey-1. The scientific workshop was non-compulsory, and all participants received the follow-up minutes from these discussions before the completion of survey-2. This participant meeting was moderated by the steering committee. The sponsor and the members of the steering committee did not participate in the surveys.

Statements which did not reach consensus in Survey-1 and any additional statements deemed appropriate from the participant meeting were included in survey-2 (*n* = 49). Survey-2 was shared electronically through individual e-mails to the participants and was completed between December 2024 and January 2025.

### Quantification and statistical analysis

A 9-point Likert scale was used to phrase the statements and rate responses as per best practice. Participants rated their level of agreement with each statement anonymously, ranging from 1 (disagreement) to 9 (agreement). These scores were divided into three groups: agree (7–9 points), neither agree nor disagree (4–6 points), and disagree (1–3 points). Open-ended and single-select radio button selection statements were quantified. Consensus was deemed to have been reached in both rounds of the survey when ≥75% of the respondents allotted either the highest or the lowest range of scores. This threshold was selected based on recommendations from previous Delphi studies and systematic reviews, which commonly report consensus levels between 70% and 80%. A 75% cut-off provides a balance between ensuring strong agreement among experts and maintaining feasibility for achieving consensus without being overly restrictive. Each question also had a ‘Do not know’ option, enabling the respondent to skip questions that were not related to their experience and ensuring that every question was viewed at least once by each respondent.

### Additional resources

This study did not utilise additional external resources.
