# Supplementary material for: Consensus recommendations for neurofibromatosis type 1 management in Asia Pacific
Source: iScience. 2026 Oct 1;29(10):117002. doi: 10.1016/j.isci.2026.117002 (PMC13657100; doi:10.1016/j.isci.2026.117002)
Supplement: Supplementary file 1 [file mmc1.pdf]

iScience, Volume *29*

Supplemental information

Consensus recommendations for neurofibromatosis type 1 management in Asia Pacific

Yemima Berman, Beom Hee Lee, Anthony Pak-Yin Liu, Yoshihiro Nishida, Ee Shien Tan, Zhichao Wang, and On behalf of APAC NF1 Expert Panel

Supplementary Material

Methods S1: Literature Search Strategy

A targeted literature review was conducted to inform the development of the Delphi questionnaire. The search was performed in the PubMed database using combinations of keywords including “Neurofibromatosis type 1”, “NF1”, “plexiform neurofibroma”, “MPNST”, “guidelines”, “consensus”, “diagnosis”, “management”, and “surveillance”, combined using Boolean operators (AND/OR).

The search included studies published up to June 2024 and was limited to English-language articles involving human subjects. Eligible study types included clinical trials, observational studies, systematic reviews, and clinical practice guidelines relevant to NF1 diagnosis, monitoring, and management.

Titles and abstracts were screened for relevance, followed by full-text review where appropriate. Additional sources were identified from reference lists and grey literature, including conference proceedings and consensus statements.

The identified evidence was synthesised qualitatively by the study team and used to generate the initial pool of Delphi statements.

No.	Query	Results
1	((neurofibromatosis 1[MeSH Terms]) OR (plexiform neurofibroma[MeSH Terms])) OR (NF1[Title/Abstract] OR NF-1[Title/Abstract] OR "NF 1"[Title/Abstract] OR "Neurofibromatosis type 1"[Title/Abstract] OR "type 1 neurofibromatosis"[Title/Abstract] OR "Neurofibromatosis 1"[Title/Abstract] OR "Neurofibromatosis-1"[Title/Abstract] OR Recklinghausen*[Title/Abstract] OR "Recklinghausen* disease"[Title/Abstract] OR "Recklinghausen* syndrome"[Title/Abstract] OR "von Recklinghausen*"[Title/Abstract] OR "Plexiform neurofibroma*"[Title/Abstract])	18,492
2	((Consensus[Title/Abstract] OR "Delphi consensus"[Title/Abstract] OR recommendation*[Title/Abstract] OR guideline*[Title/Abstract] OR management[Title/Abstract] OR diagnosis[Title/Abstract] OR surveillance[Title/Abstract] OR screening[Title/Abstract] OR epidemiology[Title/Abstract] OR prognosis[Title/Abstract] OR "disease progression"[Title/Abstract] OR "practice pattern*"[Title/Abstract] OR survival[Title/Abstract] OR mortality[Title/Abstract] OR morbidity[Title/Abstract] OR "clinical characteristic*"[Title/Abstract] OR prevalence[Title/Abstract] OR incidence[Title/Abstract] OR "Patient Journey"[Title/Abstract] OR "treatment landscape"[Title/Abstract] OR "symptom relief"[Title/Abstract] OR "Unmet needs"[Title/Abstract] OR Complication*[Title/Abstract] OR death[Title/Abstract] OR "progression of disease"[Title/Abstract] OR treatment[Title/Abstract] OR diagnostic*[Title/Abstract]) OR (clinical[Title/Abstract] AND (burden[Title/Abstract] OR manifestation[Title/Abstract] OR presentation[Title/Abstract]))) OR ((surgical[Title/Abstract] OR medical[Title/Abstract]) AND management[Title/Abstract])	12,151,502
3	#1 AND #2	8,564
4	#1 AND #2: in the last 10 years	4,113
5	#1 AND #2: in the last 10 years, Humans	2,915

6	#1 AND #2: Comment, Editorial, Letter, News	50
7	(#5) NOT (#6)	2,865
8	#7 AND (Japan OR China OR Australia OR South Korea OR Taiwan OR Hong Kong OR Singapore OR Malaysia OR Thailand)	637

Tables and figures

Table S1: Statements that achieved consensus in Survey 1

Statements	Survey 1 Consensus (%)	Survey 2 Consensus (%)
**Diagnosis**		
Q. Do you agree that six or more "typical" café-au-lait macules (CALMs), with a round or oval morphology, a homogeneous brown colour, well-defined regular borders, and a diameter larger than 5mm in the prepubertal patients serves as the initial indication for suspecting or confirming a diagnosis?	82	NR
Q. Do you agree with the following statements for NF1 associated malignant peripheral nerve sheath tumours (MPNSTs)?		
Both the surgeon and the pathologist receiving the surgical specimen should carefully document surgical margins	88	NR
Surgically resected specimens should be evaluated regarding intra-tumoral heterogeneity	80	NR
**Genetic testing and genetic counselling**		
Q. Do you agree that the role of genetic counsellors is crucial to address the challenges faced during selecting the most appropriate genetic testing based on the clinical scenario, interpreting genetic testing results including the implications of	90	NR

negative results, and providing accurate recurrence risk information to patients?		
Q. When the phenotype cannot be differentiated from other disorders, do you agree that a multigene panel containing NF1, SPRED1, piebaldism (KIT, KITLG), mismatch repair (MLH1, MSH6, PMS2, MSH2) and other relevant genes may be considered?	89	NR
Q. Considering various techniques like multiplex ligation-dependent probe amplification (MLPA), microarray analysis, and optical genome mapping (OGM) available for detecting gene loss, do you agree that additional investigation for potential gene deletion should be entertained if the initial sequencing results indicate no mutations?	78	NR
Q.* Do you agree that when performing genetic testing for NF1, concurrent genetic testing of the following differential diagnosis should be considered?		
Legius syndrome	88	NR
Constitutional mismatch repair deficiency (CMMRD) syndrome	75	NR
Q. Do you agree with the following points for couples considering preimplantation genetic testing (PGT) because of NF1:		
Timely reproductive PGT-related counselling is important	84	NR

PGT-related counselling should include all reproductive options available to the couple, enabling them to reach their own conclusion about the suitability of treatment.	81	NR
Several couples cannot pursue PGT due to factors like maternal age, diminished ovarian reserve, de novo inheritance or the inability to detect an underlying genetic defect.	76	NR
Q. Do you agree that NF1 individuals with a missense variant involving one of the codons from 844–848 have a higher prevalence of plexiform neurofibromas, spinal neurofibromas, optic pathway gliomas, and skeletal abnormalities?	78	NR
**Clinical manifestation and monitoring**		
Q. Do you agree with the proposed multidisciplinary approach for the care of NF1 patients?		
NF1-specialized centres should monitor complex cases (such as multiple skin tumours as lumps under the skin, malignancy)	98	NR
Q. Do you think that dermatologists play an important role, especially with grade 3 rashes being common, when considering starting treatment of NF1-plexiform neurofibroma in both children and adults with MEK inhibitors?	84	NR
Q. Do you agree with the following surveillance protocol for children with NF1?		
Physical examinations at least twice a year until age five and then at least once a year after that.	88	NR
Annual neurologic examination for headaches and seizures.	85	NR

Child's head circumference should be checked regularly.	93	NR
When child reaches school age, doctors should recommend screening for learning disorders and attention deficit hyperactivity disorders.	95	NR
Annual review should include a pain score assessment for children.	85	NR
Annual cardiac assessment through evaluating blood pressure and/or echocardiography.	80	NR
Careful examination looking for scoliosis and/or limb asymmetry.	95	NR
Check for accelerated growth or precocious puberty.	88	NR
Q. Do you agree that MRI is preferred over CT scanning to minimize ionizing radiation exposure in patients with NF1?	98	
Q. Do you agree that NF1-plexiform neurofibromas have the highest probability of developing into malignant peripheral nerve sheath tumours (MPNSTs) when compared to other types of neurofibromas?	94	
Q. Do you agree with the following statements related to NF1-plexiform neurofibromas?		
NF1-plexiform neurofibroma with rapid growth (>20% increase in volume per year) and impending morbidity is likely a candidate for intervention	89	

A slowly growing NF1-plexiform neurofibroma (<5% increase in volume per year), without actual or impending morbidity may be suitable for observation	80	
Management of NF1-plexiform neurofibroma depends on location of the tumour, clinical symptoms and patients desire for treatments	91	
Q. Do you agree with the following statements for children and adult patients with NF1-plexiform neurofibroma?		
Radiotherapy is not recommended due to the increased risk of secondary malignancy and Moyamoya syndrome	85	NR
MEK inhibitors (e.g. selumetinib) can be considered as treatment option for symptomatic plexiform neurofibroma	91	NR
MEK inhibitors (e.g. selumetinib) can be considered if the NF1-plexiform neurofibroma remains symptomatic following partial resection	91	NR
Considering the impact of visible manifestations in NF1-plexiform neurofibroma, psychological support should be offered	96	NR
Q. Do you agree that radiotherapy to the brain should be avoided when at all possible, for children with NF1 because of the risk of moyamoya syndrome and secondary tumours?	90	NR
Q. Do you agree it is important to discuss the goals and expected outcomes (including timeframe) with the NF1-	93	NR

plexiform neurofibroma patient/caregiver before any intervention, like surgery or MEK inhibitor treatment?		
Q. Do you agree with the following statements for patients with NF1 associated cutaneous neurofibroma?		
Clinical assessment consisting of visual inspection and palpation should be repeated at every clinical visit	89	NR
Patients should be offered psychological support	90	NR
Q. Do you agree with the following statements in children without known optic pathway glioma (OPG)?		
Ophthalmological assessment should be repeated annually until the age of 8 years	89	NR
Formal annual visual screening is advised in children >8 years until adulthood	76	NR
Q.* In relation to bone health of NF1 patients, do you agree with the following points?		
Monitoring for curve progression for management of scoliosis	92	NR
Annual clinical evaluation of the back with Adam's forward bend test in case of any concern related to scoliosis	82	NR
Q.* Do you agree with the following statements for breast cancer screening of women with NF1?		
Annual screening in NF1 should begin as soon after the age of 30 years.	80	NR

Q. Do you agree that the management of cognitive problems and behavioural difficulties in childhood NF1 patients may include:		
A special educational needs coordinator may be involved at an early stage	82	NR
Close liaison between teachers, educational psychologists, occupational therapists, and community paediatricians	88	NR
Cognitive behavioural therapy	81	NR
Specific focus on executive functioning assessment	78	NR
Support with study skills and time management	82	NR
Q. Do you agree with the following statements for NF1 associated pheochromocytoma patients?		
Pheochromocytoma should be considered in hypertensive NF1 patients who are over 30 years of age, pregnant, and/or have paroxysmal hypertension, hypertension-associated headache, palpitations, or sweating.	82	NR
Q. In NF1-associated gastrointestinal stromal tumour (GIST) patients, do you agree that resection should be considered for tumours that are large (>2cm) or causing symptoms, due to a risk of bleeding, rupture, and potential malignancy with metastasis?	81	NR
**Child Vs Adult transition**		

Q. Do you agree that to prevent NF1 patients from being lost to follow-up, it's crucial to plan the transition from paediatric to adult care in advance and discuss this topic with patients/caregivers?	92	NR
Q. Do you agree that patients with NF1-associated malignant peripheral nerve sheath tumours (MPNSTs) or atypical neurofibromatosis neoplasm with uncertain biologic potential (ANNUBPS) should undergo a baseline whole-body MRI, at the transition age?	85	NR
Q. Do you agree that most severe skeletal manifestations, such as long bone dysplasia/pseudarthrosis, dystrophic scoliosis, and sphenoid wing dysplasia, often manifest during childhood but can have enduring effects into adulthood?	89	NR
Q. Due to the risk of tumour complications in NF1, experts recommend regular check-ups by NF1 specialists, with at least a minimum of:<br>• annually in children up to 10 years,<br>• once every two years in children older than 10 years and<br>• once every 3 years in adults.<br>Do you agree that transition from adolescence to adulthood require more frequent systematic clinical assessments compared to those mentioned above.	83	NR
Q. Do you agree with the following statements for patients with NF1-plexiform neurofibromas (NF1-PN) transitioning from childhood to adulthood?		

Whole-body MRI imaging should be undertaken at the transition to evaluate internal tumour burden as a predictor for the development of NF1-PN or malignant peripheral nerve sheath tumours (MPNSTs) risk	77	NR
*Question is modified in survey 2		

Table S2: Statements that did not achieve consensus in Survey 1 and achieved consensus in Survey 2

Statements	Survey 1 Consensus (%)	Survey 2 Consensus (%)
**Diagnosis**		
Q.* Do you agree that classification of NF1-plexiform neurofibroma on MRI should include the following?		
* A) Morphology or internal structure: Diffuse, Fascicular/multi-nodular, Predominantly diffuse or fascicular/multi-nodular, Mixed<br>B) Depth: Deep, Superficial, Predominantly deep or superficial, Mixed<br>C) Relationship to adjacent tissues:<br>i) Interdigitating (Tumours with ill-defined borders which penetrate and cannot be easily separated from adjacent tissues<br>ii) Circumscribed (Tumours with smoothly defined borders which may compress and/or displace adjacent structures<br>iii) Predominantly interdigitating or circumscribed<br>iv) Mixed	NR	85
* Additionally, do you agree that NF1-plexiform neurofibroma can be classified as "Distinct nodular lesion" to describe peripheral nerve sheath tumors with a characteristic MRI appearance?	48	76
Q. Do you agree with the following statements for NF1-associated malignant peripheral nerve sheath tumours (MPNSTs)?		
* If the final pathology assessment indicates positive margins (excluding bone, nerves, or major vessels), considering that achieving negative margins is the goal for patients with MPNST, a thoughtful consideration should be given to conducting another surgical resection to ensure negative margins, as long as there is no significant impact on function	64	85
Adjuvant radiotherapy should be considered for cases where margins are close or there are microscopically positive margins involving bone, vital vessels, and nerves	59	75
**Genetic testing and genetic counselling**		
Q.* Do you agree that when performing genetic testing for NF1, concurrent genetic testing of the following differential diagnosis should be considered?		

RASopathy syndromes (e.g. Noonan syndrome)	73	84
Do you agree with the following for couples considering preimplantation genetic testing (PGT) because of NF1:		
* PGT is a suitable option for couples wishing to select embryos unaffected with NF1	63	75
**Clinical manifestation and monitoring**		
Q. Do you agree with the proposed multidisciplinary approach for the care of NF1 patients?		
# The definition of non-complex cases, "Patients with an NF1 diagnosis, with no PN or internal neurofibroma, no pain, no growth issues, and no specific symptoms can be considered to be non-complex cases"?	NA	93
* Non-complex cases can be monitored locally by general practitioners/paediatricians who should be given appropriate guidelines for the management of NF1?	73	88
Q.* Do you agree to consider whole-body MRI, if available, for evaluating patients with NF1, given the increased risk of malignant peripheral nerve sheath tumours (MPNSTs) being associated with a high internal tumour burden in specific cases, such as at the age when MPNST is likely to develop, or during the transition from childhood to adulthood ?	NR	88
Q # The frequency and type of radiological assessment will depend on a number of factors such as size, location of PN, age of patient (sedation requirements), other symptoms which may indicate malignant transformation. (this blue note is applicable to all three options asked below)		
# Do you agree that if the patient suffers from a very large extensive plexiform neurofibroma, they should receive whole-body MRI once a year?	NA	75
# Do you agree that if the region of the affected plexiform neurofibroma can be covered by localized MRI, then it can be followed up by localized MRI once a year?	NA	77
# Do you agree to perform F18-FDG-PET scan in patients upon suspicion of malignant transformation?	NA	88
Q. Do you agree with the following statements for children and adult patients with NF1-plexiform neurofibroma?		
# In some cases, partial resection may be very beneficial for patients	NA	79
Q. Do you agree with the following statement for patients with NF1 associated cutaneous neurofibroma?		

a)* Removal could be by laser, surgery, electrodesiccation, radiofrequency ablation or, if available, topical treatment (e.g. topical MEK inhibitor).<br>Note: All the above mentioned treatments are at the same level and not sequential	57	89
Q.* In relation to bone health of NF1 patients, do you agree with the following points?		
d) Treatment for NF1 associated osteoporosis should be same as recommended for the general population	73	89
Q.* Do you agree with the following statements for breast cancer screening of women with NF1?		
Screening with annual breast MRI should be the primary approach.	62	77
* 3D tomosynthesis mammography combined with Ultrasound is the second best alternative when MRI is not available	69	93
* Annual breast cancer screening for NF1 should continue until 50 years	63	85
Risk-reducing bilateral mastectomy for woman without breast cancer should not be performed in NF1 patients	72	91
Q. Do you agree that the management of cognitive problems and behavioural difficulties in childhood NF1 patients may include:		
* Pharmacological intervention for ADHD under experienced supervision	59	86
Q.* Do you agree with the following statement on pheochromocytoma screening in patients with NF1		
* Routine biochemical screening is recommended for hypertensive NF1 patients	NR	86
Q.* Do you agree with the following recommended treatment options for NF1 patients with symptomatic optic pathway gliomas (OPGs)?		
Vincristine and Carboplatin as first-line treatment (standard of care).	64	84

Q. Patients with NF1 typically do not show KIT proto-oncogene (KIT) or platelet-derived growth factor receptor α (PDGFRA) overexpression in gastrointestinal stromal tumours (GIST). Do you agree that this will restrict the utility of imatinib (tyrosine kinase inhibitor) in this population?	57	79
**Child Vs Adult transition**		
Q. Do you agree with the following statement for patients with NF1-plexiform neurofibromas (NF1-PN) transitioning from childhood to adulthood?		
* If available, Whole-body MRI assessment at higher frequency may be considered for patients at high risk for malignant peripheral nerve sheath tumours(MPNSTs)	72	88
*Question is modified in survey 2<br># New question added in survey 2		

Figure S1: Survey-2 non-consensus statements for Diagnosis

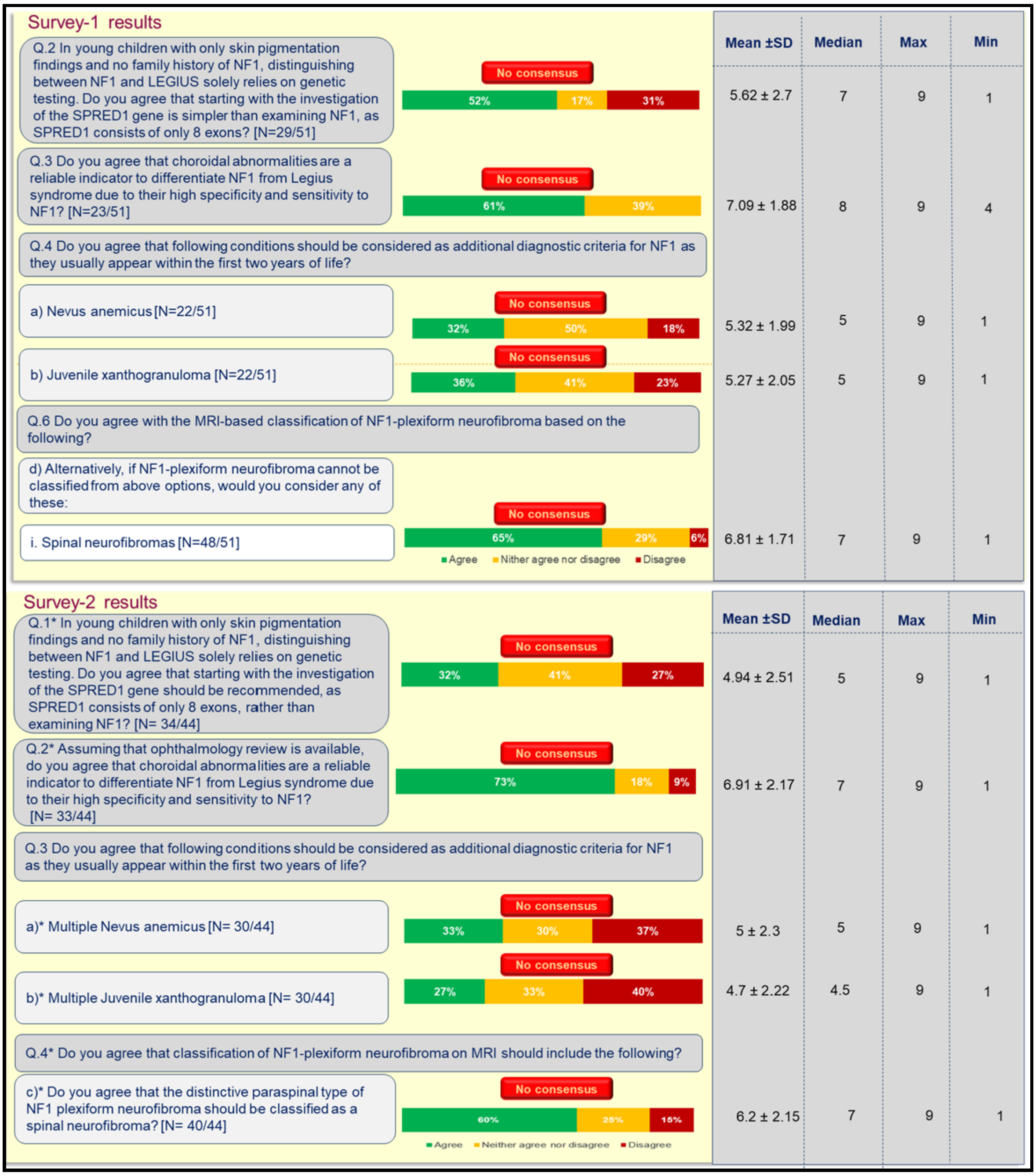

*Question is modified in survey 2

Figure S2: Survey-2 non-consensus statements for Genetic testing and genetic counselling

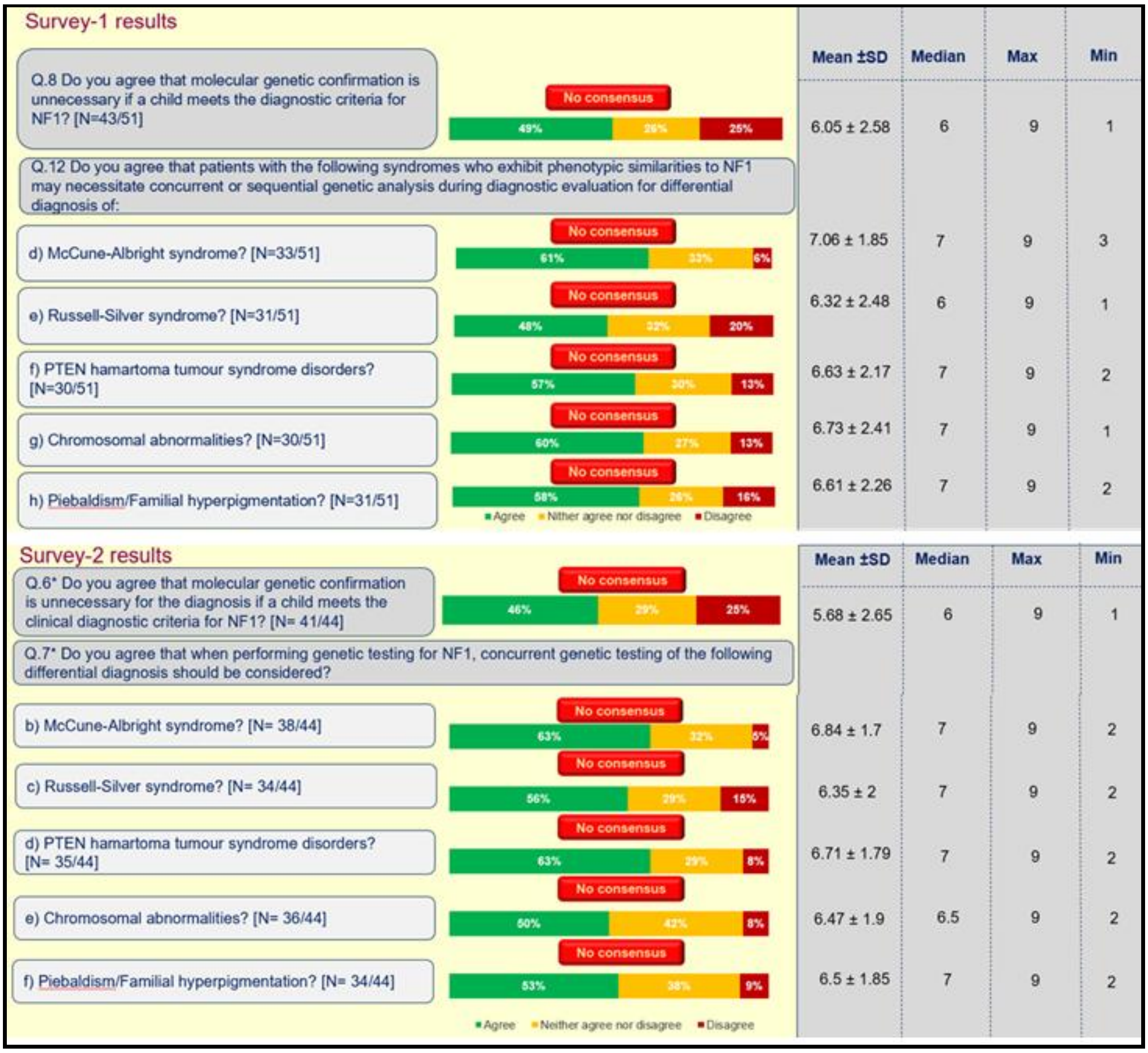

*Question is modified in survey 2

Figure S3: Survey-2 non-consensus statements for Clinical manifestation and Monitoring

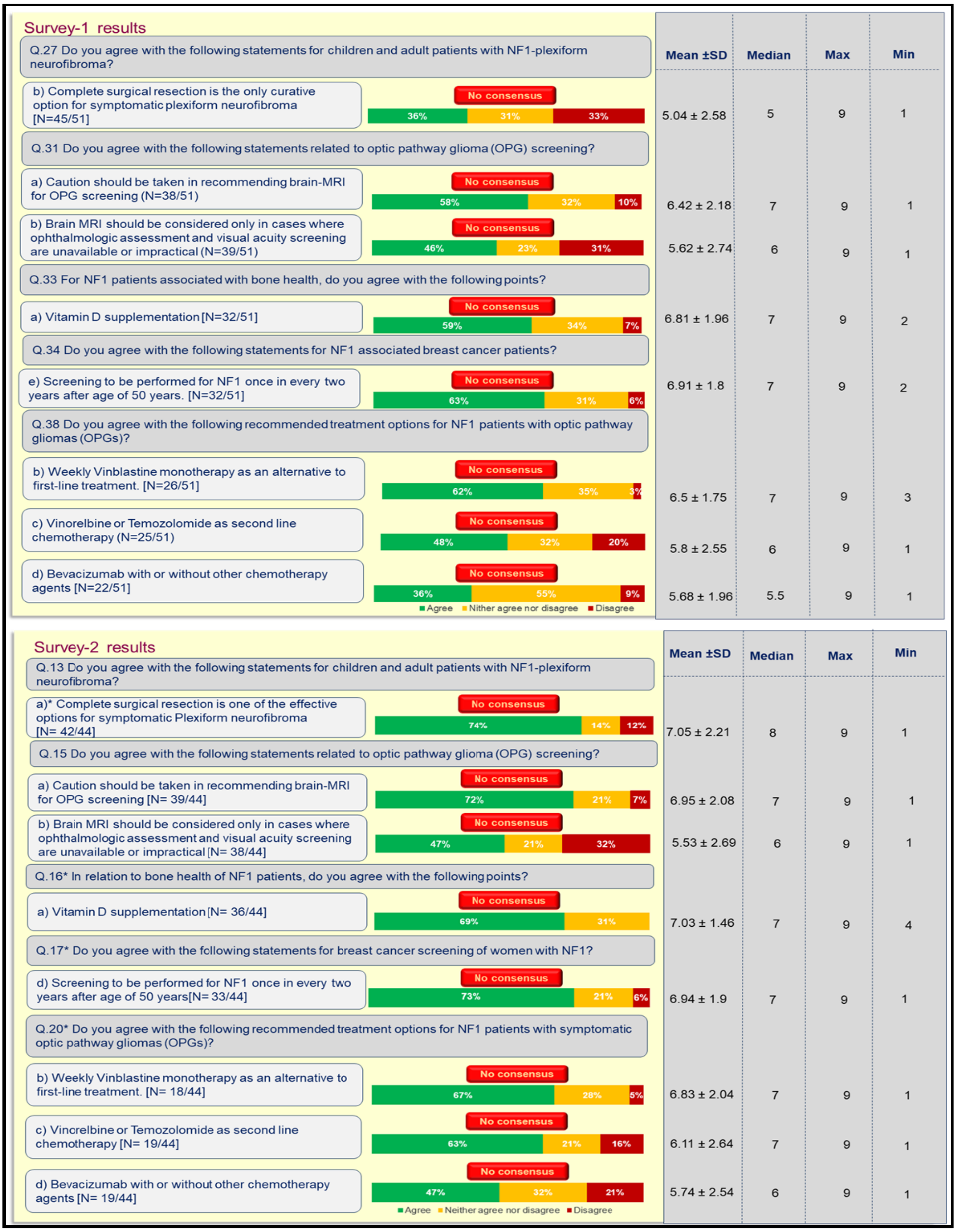

*Question is modified in survey 2

Figure S4: Survey-2 non-consensus statements for Child vs. Adult transition

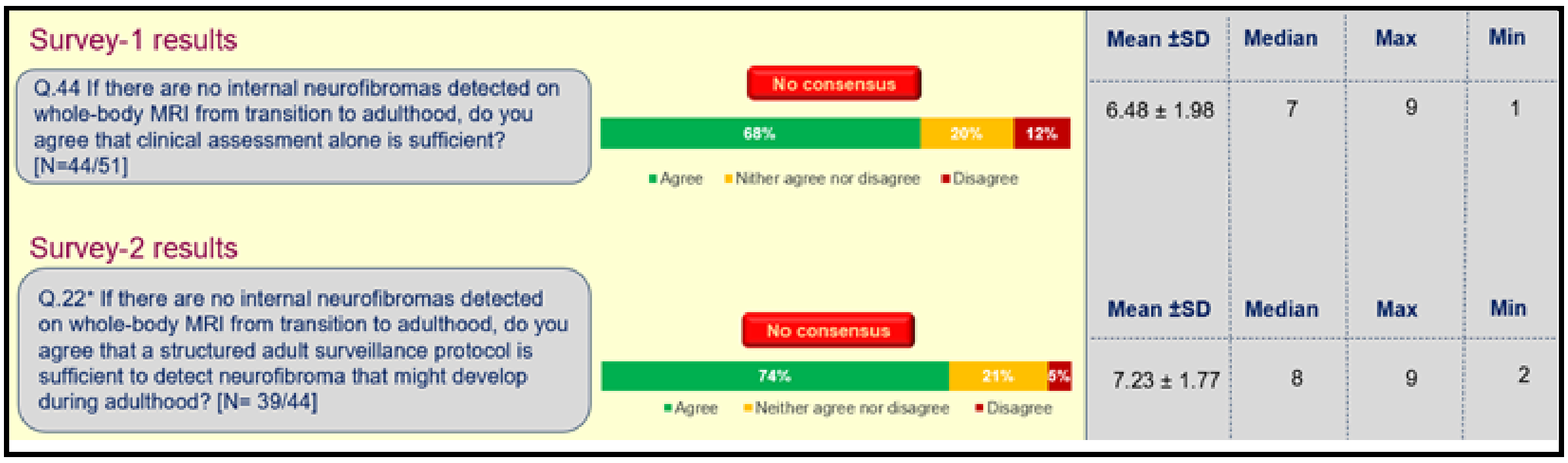

*Question is modified in survey 2

Figure S5: Components of multidisciplinary team

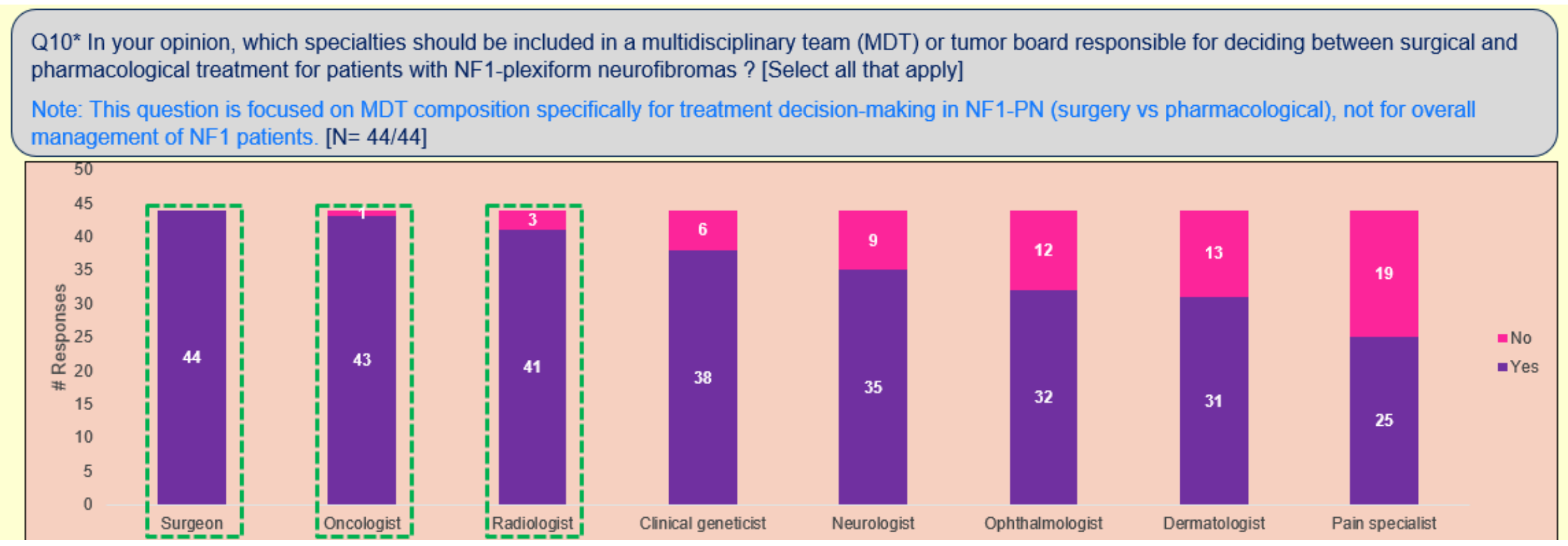

Figure S6

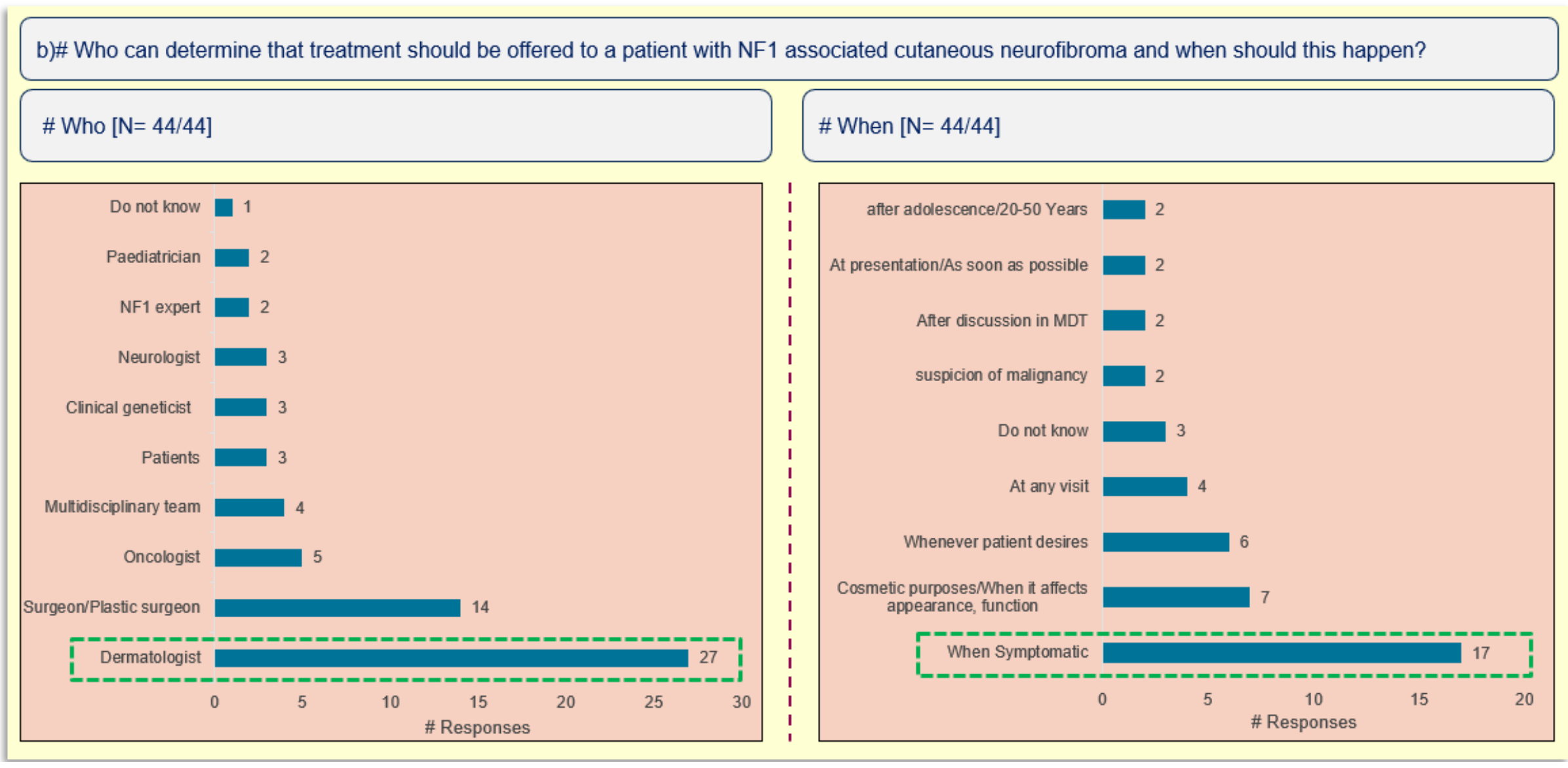

Figure S7

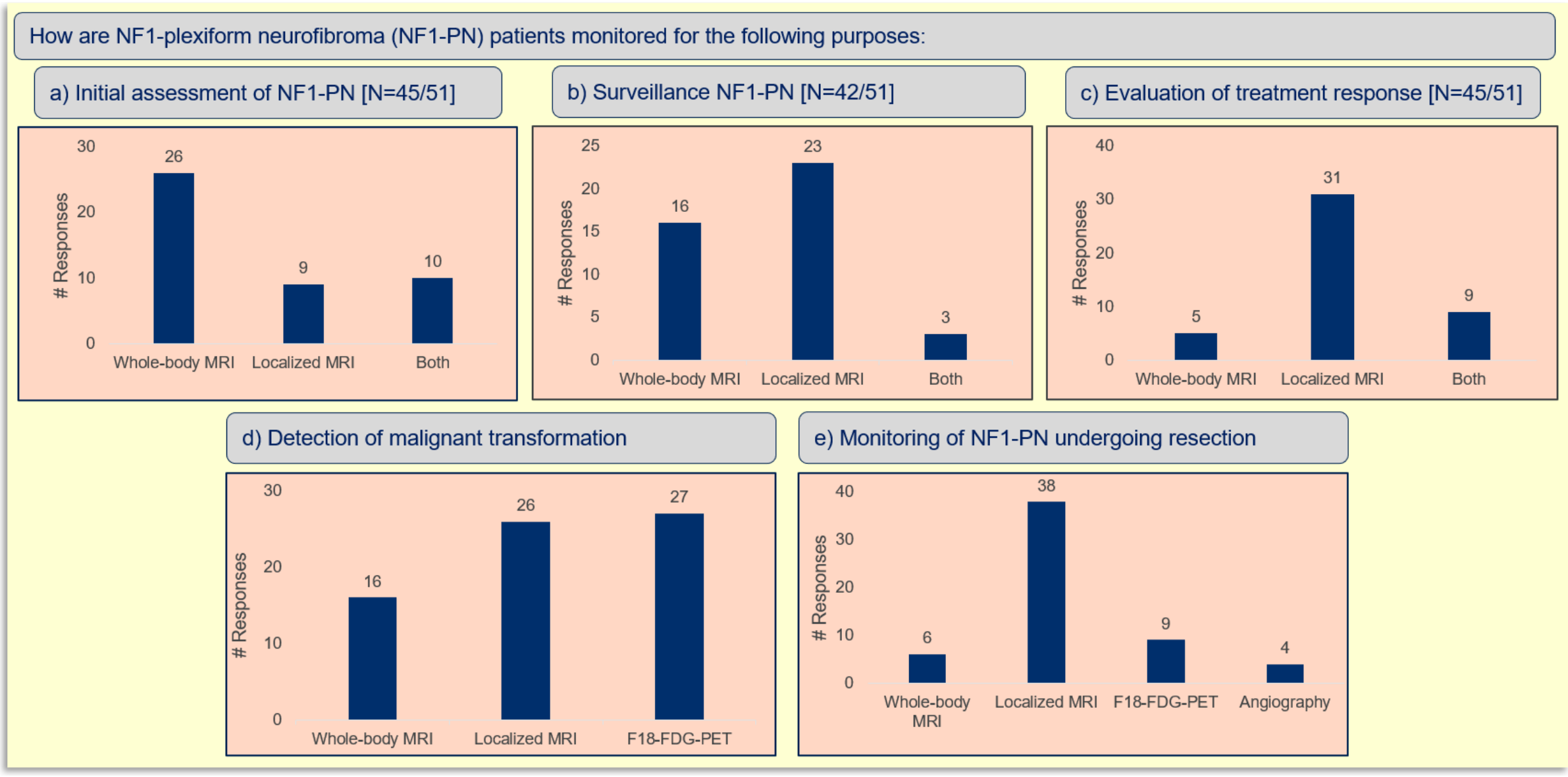

Figure S8

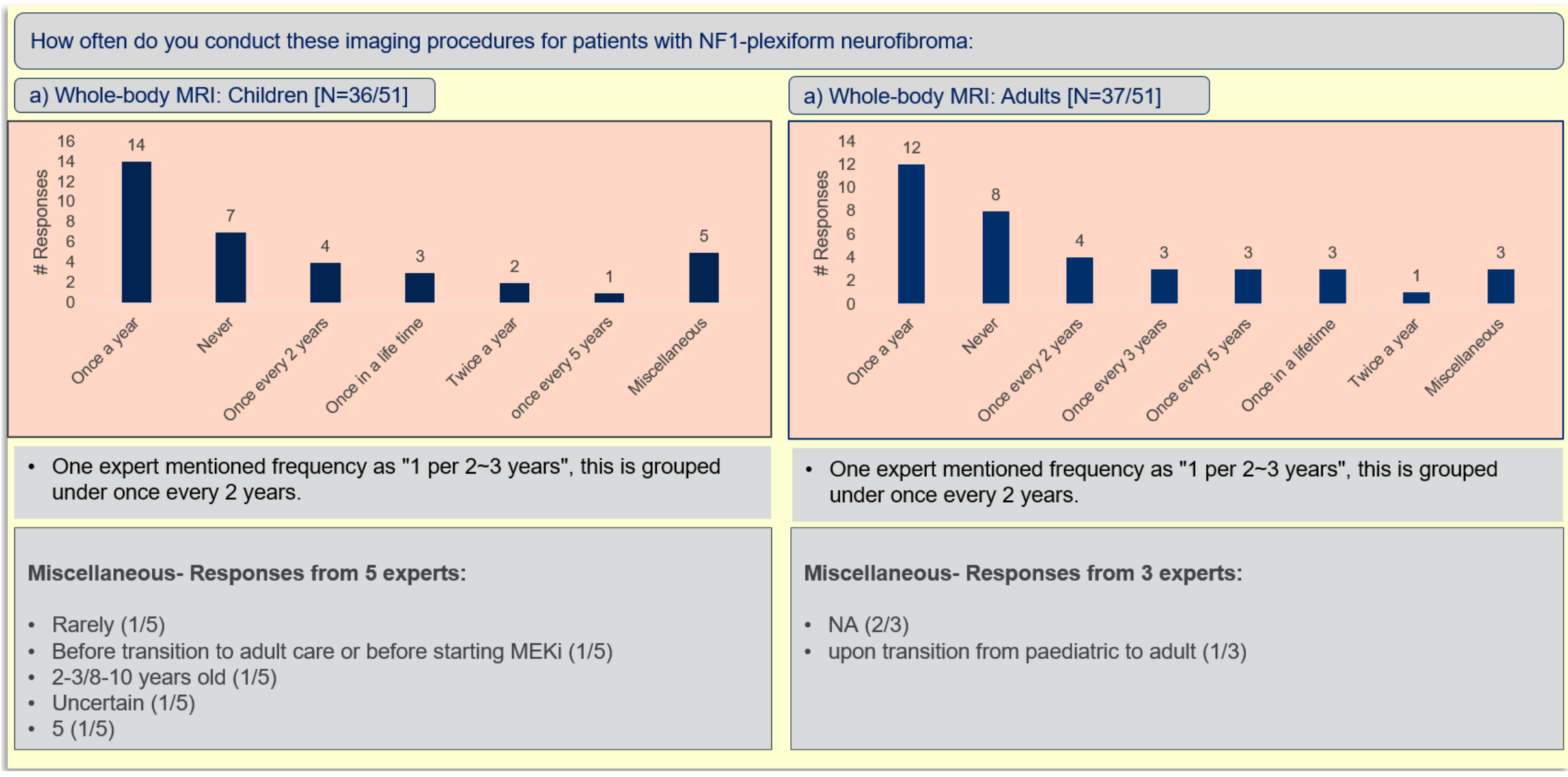

How often do you conduct these imaging procedures for patients with NF1-plexiform neurofibroma:

b) Localized MRI: Children [N=35/51]

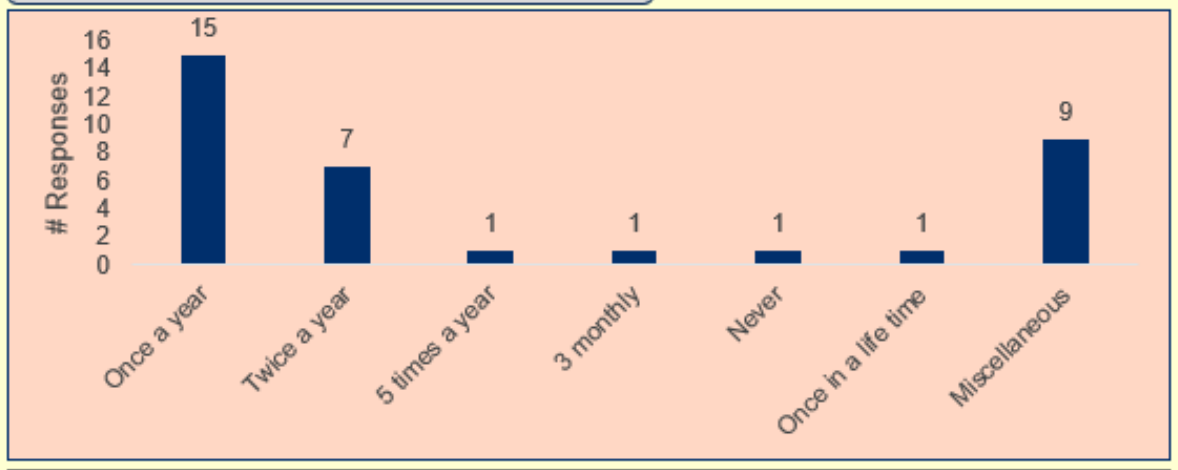

Miscellaneous- Responses from 9 experts:

- 4 (1/9)
- No specific time. Do when suspicious of malignant transformation and monitoring of treatment response 6 monthly x2 then yearly or when any signs and symptoms occur. (1/9)
- If necessary after WB MRI (1/9)
- Depends on location, more frequent if growing or on treatment, otherwise 1-2yearly to monitor deep-seated lesions (1/9)
- When MPNST is suspected or vital organs are involved/compressed (1/9)
- Rarely performed (1/9)
- When needed(large tumor, perispinal tumor, tumor increasing, etc) (1/9)
- Uncertain (1/9)
- 5 (1/9)

b) Localized MRI: Adults [N=35/51]

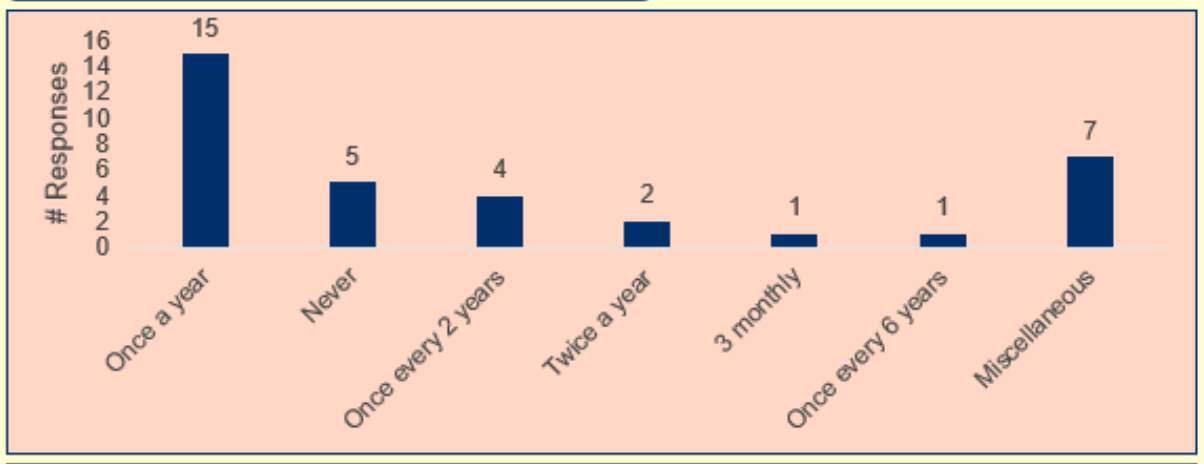

Miscellaneous- Responses from 7 experts:

- NA (2/7)
- No specific time. Do when suspicious of malignant transformation, and monitoring of treatment response 6 monthly x2 then yearly or when any signs and symptoms occur. (1/7)
- if necessary after WB MRI (1/7)
- when MPNST is suspected or vital organs are involved/compressed (1/7)
- rarely performed (1/7)
- when needed (1/7)

How often do you conduct these imaging procedures for patients with NF1-plexiform neurofibroma:

c) F18-FDG-PET scan: Children [N=24/51]

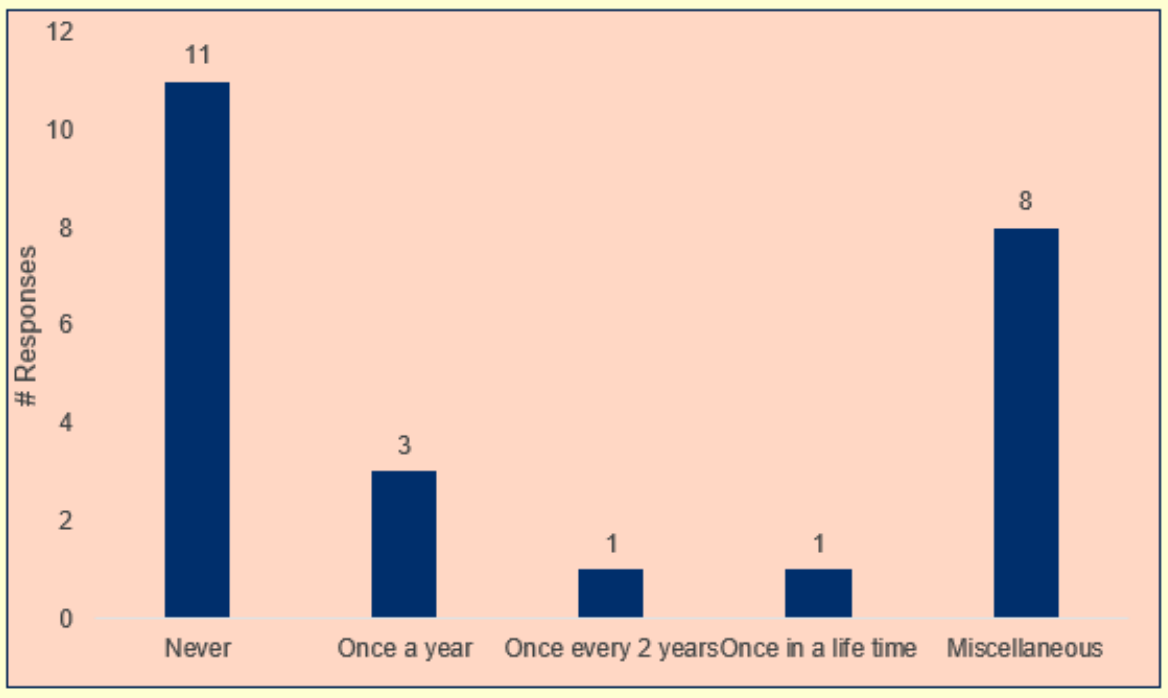

Miscellaneous- Responses from 8 experts:

- Only in case of suspicious malignancy (3/8)
- In the case of suspected MPNST (2/8)
- Only when needed (2/8)
- If necessary after WB MRI (1/8)

c) F18-FDG-PET scan : Adults [N=25/51]

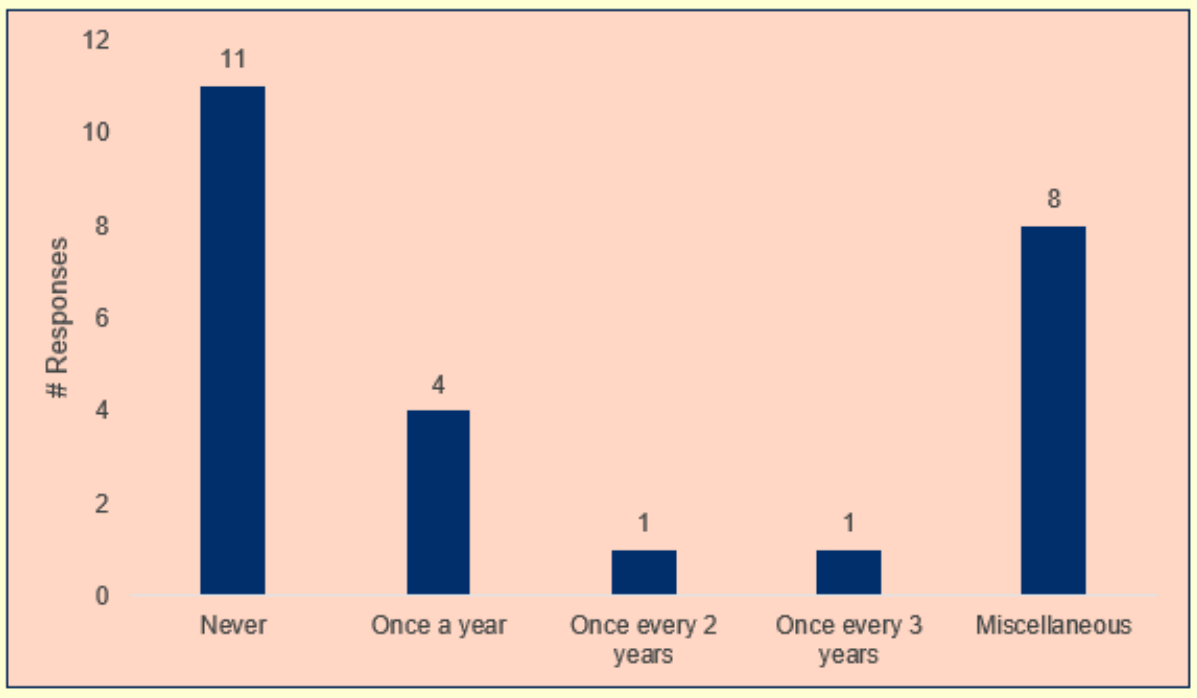

Miscellaneous- Responses from 8 experts:

- Only in case of suspicious malignancy (2/8)
- In the case of suspected MPNST (2/8)
- NA (2/8)
- Only when needed (1/8)
- If necessary after WB MRI (1/8)

Figure S9

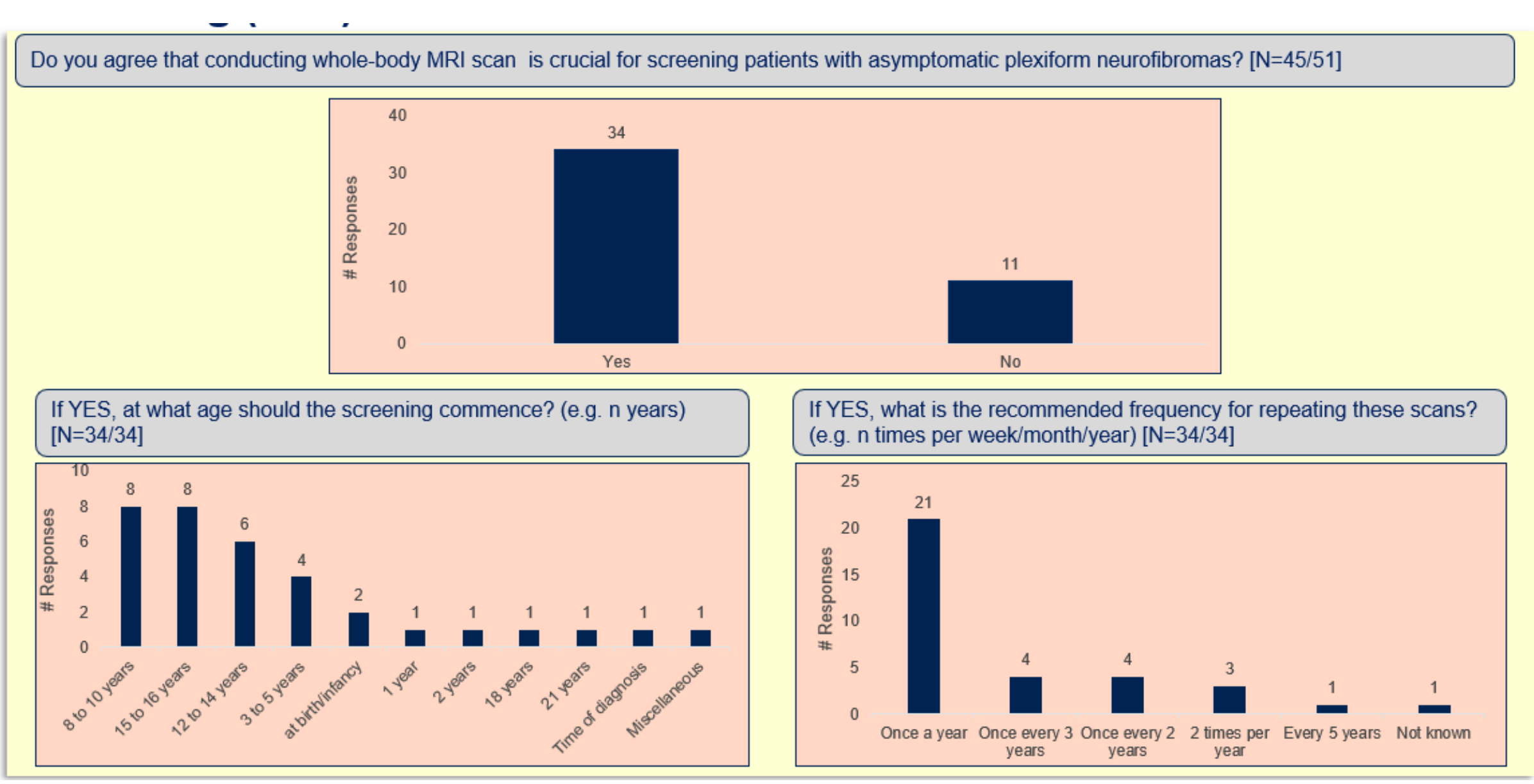

Figure S10

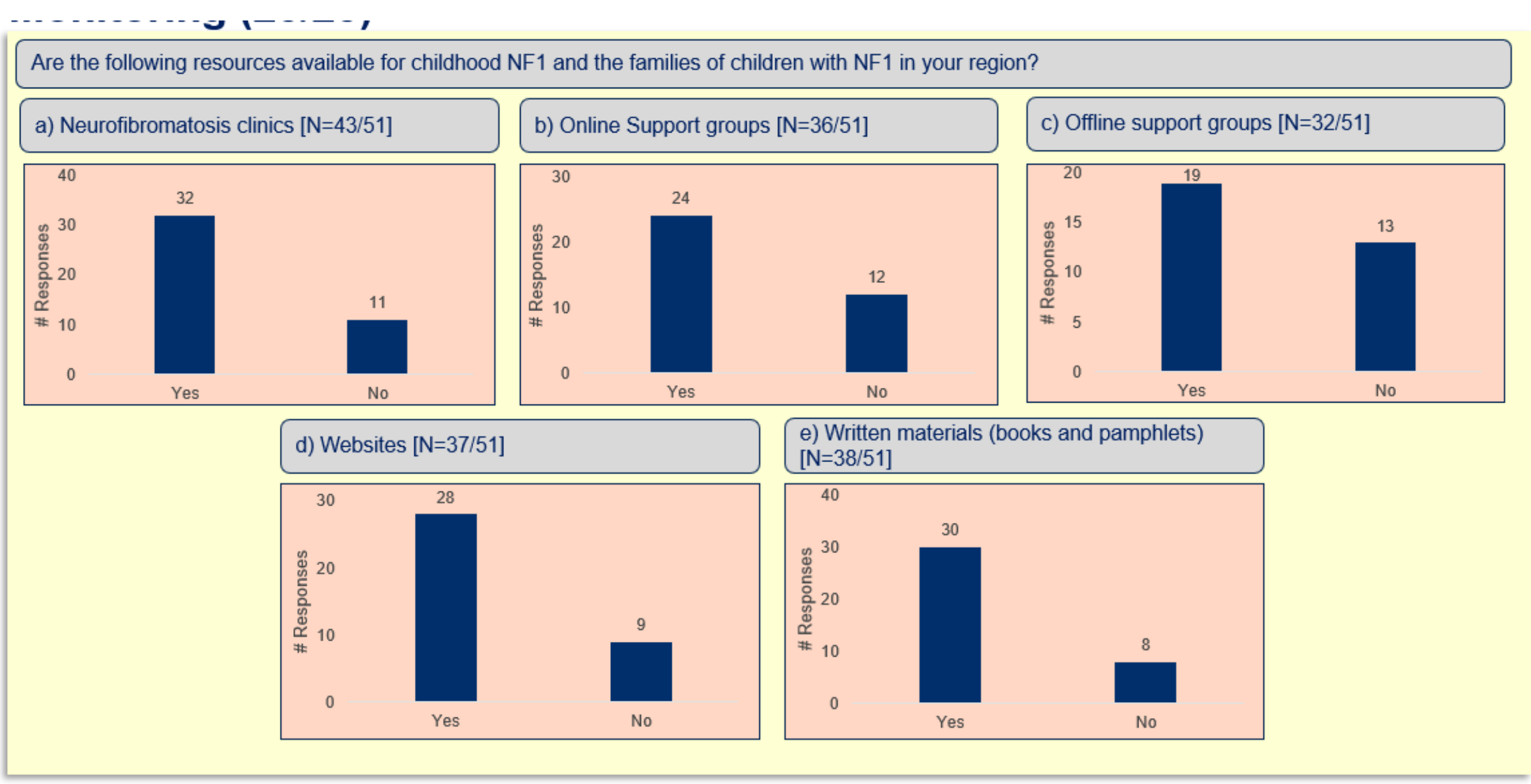

Figure S11

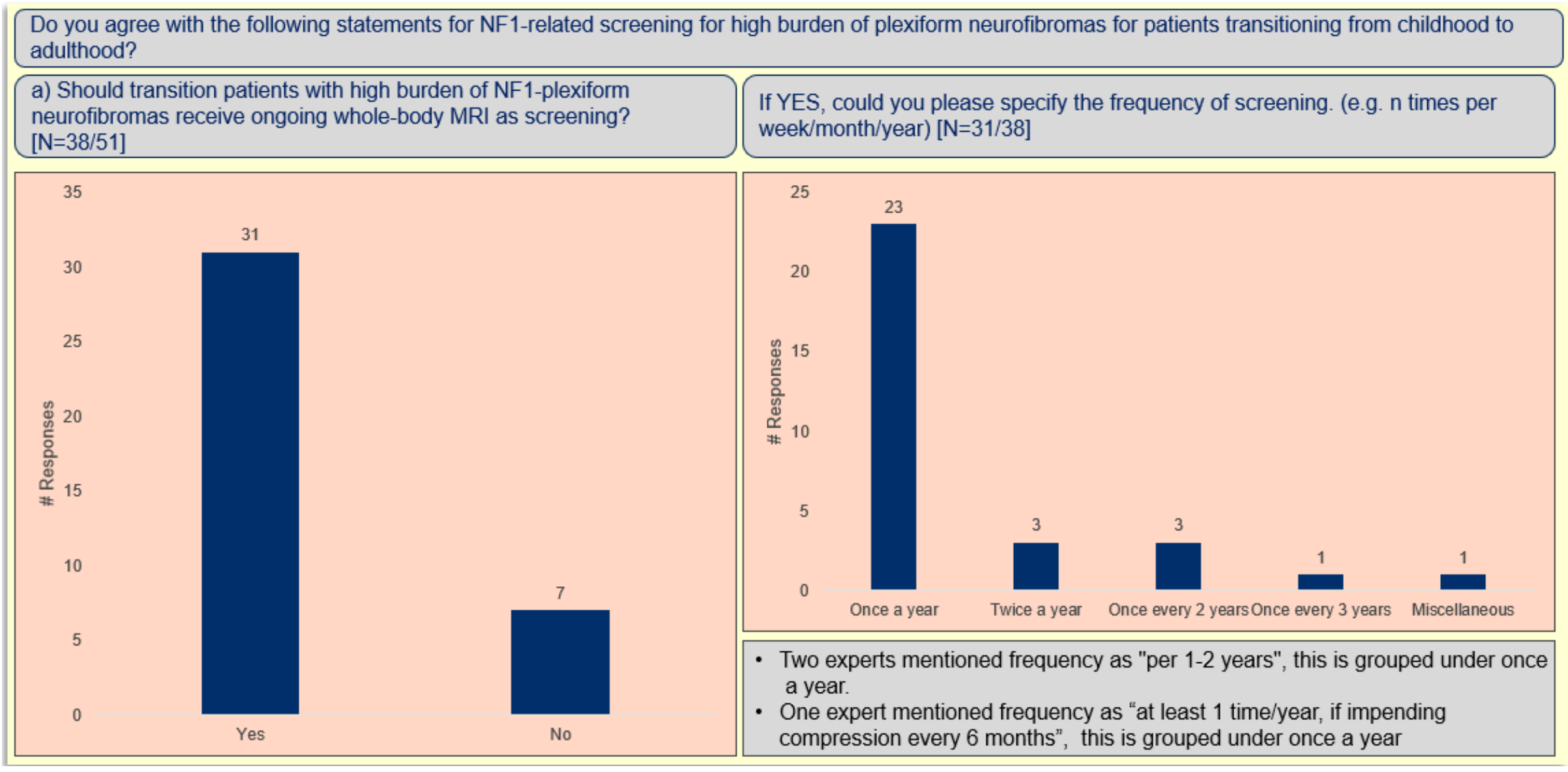
Do you agree with the following statements for NF1-related screening for high burden of plexiform neurofibromas for patients transitioning from childhood to adulthood?
a) Should transition patients with high burden of NF1-plexiform neurofibromas receive ongoing whole-body MRI as screening? [N=38/51]
If YES, could you please specify the frequency of screening. (e.g. n times per week/month/year) [N=31/38]
Responses
35
30
25
20
15
10
5
0
31
7
Yes
No
Responses
25
20
15
10
5
0
23
3
3
1
1
Once a year
Twice a year
Once every 2 years
Once every 3 years
Miscellaneous
• Two experts mentioned frequency as "per 1-2 years", this is grouped under once a year.
• One expert mentioned frequency as "at least 1 time/year, if impending compression every 6 months", this is grouped under once a year

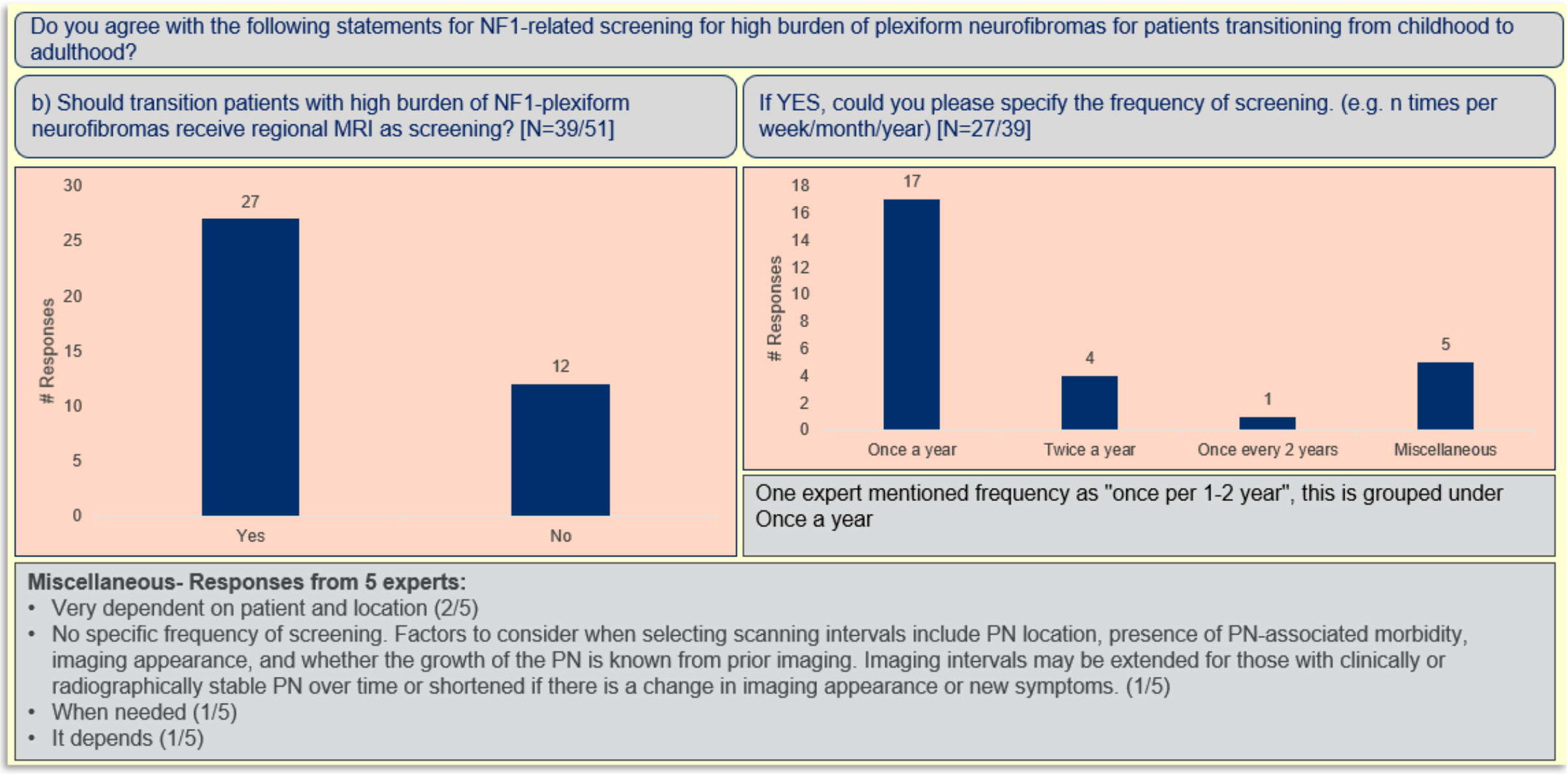
Do you agree with the following statements for NF1-related screening for high burden of plexiform neurofibromas for patients transitioning from childhood to adulthood?
b) Should transition patients with high burden of NF1-plexiform neurofibromas receive regional MRI as screening? [N=39/51]
If YES, could you please specify the frequency of screening. (e.g. n times per week/month/year) [N=27/39]
Responses
30
25
20
15
10
5
0
27
12
Yes
No
Responses
18
16
14
12
10
8
6
4
2
0
17
4
1
5
Once a year
Twice a year
Once every 2 years
Miscellaneous
One expert mentioned frequency as "once per 1-2 year", this is grouped under Once a year
Miscellaneous- Responses from 5 experts:
• Very dependent on patient and location (2/5)
• No specific frequency of screening. Factors to consider when selecting scanning intervals include PN location, presence of PN-associated morbidity, imaging appearance, and whether the growth of the PN is known from prior imaging. Imaging intervals may be extended for those with clinically or radiographically stable PN over time or shortened if there is a change in imaging appearance or new symptoms. (1/5)
• When needed (1/5)
• It depends (1/5)

Do you agree with the following statements for NF1-related screening for high burden of plexiform neurofibromas for patients transitioning from childhood to adulthood?

c) Should FDG PET be used as a screening tool for transition patients with high burden of NF1-plexiform neurofibromas? [N=37/51]

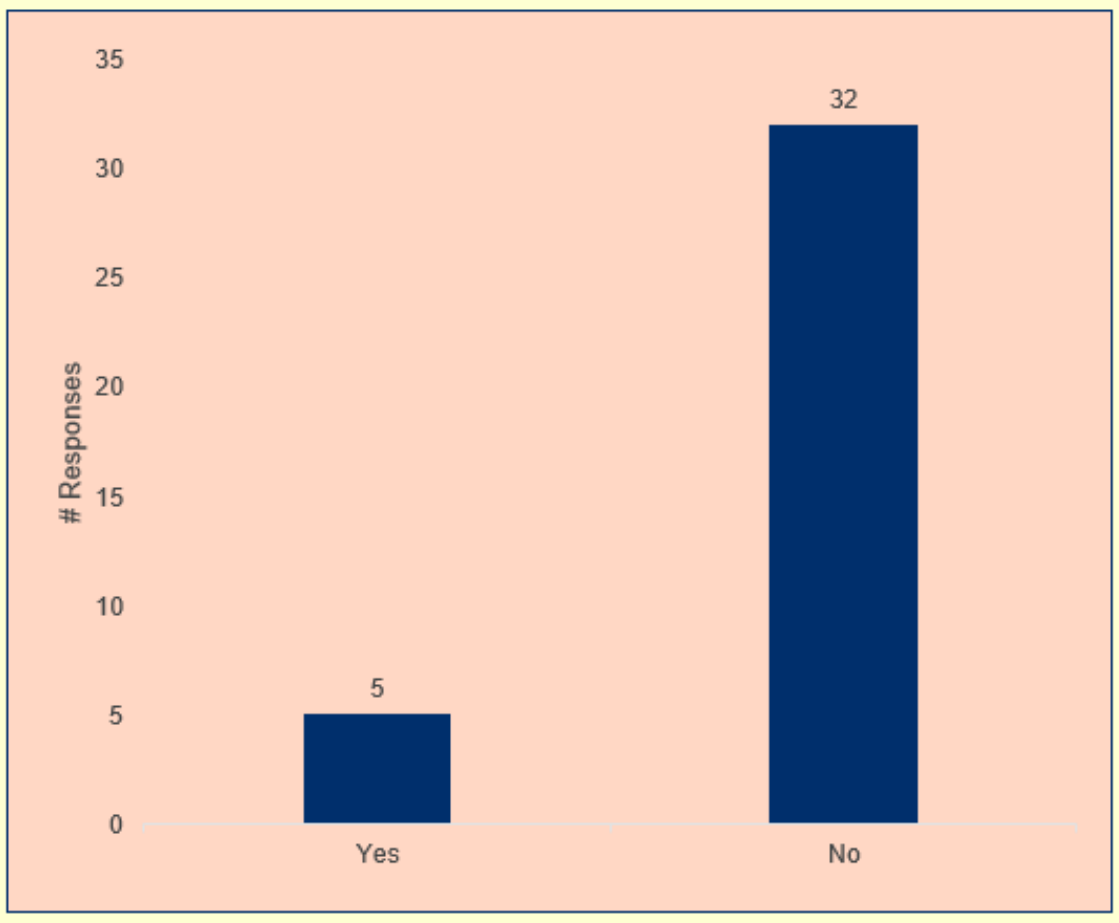

If YES, could you please specify the frequency of screening. (e.g. n times per week/month/year) [N=5/37]

Responses from 5 experts:

- Once (1/5)
- If an MRI is not available. No specific frequency of screening. Factors to consider when selecting scanning intervals include PN location, presence of PN-associated morbidity, imaging appearance, and whether the growth of the PN is known from prior imaging. Imaging intervals may be extended for those with clinically or radiographically stable PN over time or shortened if there is a change in imaging appearance or new symptoms. (1/5)
- At transition, when MPNST is suspected on whole-body MRI (1/5)
- Suspicion of atypical of malignancy (1/5)
- Once a year (1/5)
